# Obesity and exercise training alter inflammatory pathway skeletal muscle small extracellular vesicle microRNAs

**DOI:** 10.1113/EP090062

**Published:** 2022-04-03

**Authors:** Brian P. Sullivan, Yaohui Nie, Sheelagh Evans, Chris K. Kargl, Zach R. Hettinger, Ron T. Garner, Monica J. Hubal, Shihuan Kuang, Julianne Stout, Timothy P. Gavin

**Affiliations:** ^1^ Department of Health and Kinesiology Purdue University West Lafayette Indiana USA; ^2^ Department of Animal Sciences Purdue University West Lafayette Indiana USA; ^3^ College of Science and Humanities Husson University Bangor Maine USA; ^4^ Department of Kinesiology Indiana University–Purdue University Indianapolis Indianapolis Indiana USA; ^5^ Department of Pediatrics Indiana University School of Medicine–West Lafayette West Lafayette Indiana USA

**Keywords:** exercise training, extracellular vesicles, inflammation, microRNA, obesity

## Abstract

**New Findings:**

- **What is the central question of this study?**
  Is 1 week of exercise training sufficient to reduce local and systemic inflammation?
  Do obesity and short‐term concurrent aerobic and resistance exercise training alter skeletal muscle extracellular vesicle (EV) contents?
- **What is the main finding and its importance?**
  Obesity alters skeletal muscle small EV microRNAs targeting inflammatory and growth pathways. Exercise training alters skeletal muscle small EV microRNAs targeting inflammatory pathways, indicative of reduced inflammation. Our findings provide support for the hypotheses that EVs play a vital role in intercellular communication during health and disease and that EVs mediate many of the beneficial effects of exercise.

**Abstract:**

Obesity is associated with chronic inflammation characterized by increased levels of inflammatory cytokines, whereas exercise training reduces inflammation. Small extracellular vesicles (EVs; 30–150 nm) participate in cell‐to‐cell communication in part through microRNA (miRNA) post‐transcriptional regulation of mRNA. We examined whether obesity and concurrent aerobic and resistance exercise training alter skeletal muscle EV miRNA content and inflammatory signalling. Vastus lateralis biopsies were obtained from sedentary individuals with (OB) and without obesity (LN). Before and after 7 days of concurrent aerobic and resistance training, muscle‐derived small EV miRNAs and whole‐muscle mRNAs were measured. Pathway analysis revealed that obesity alters small EV miRNAs that target inflammatory (SERPINF1, death receptor and Gα_i_) and growth pathways (Wnt/β‐catenin, PTEN, PI3K/AKT and IGF‐1). In addition, exercise training alters small EV miRNAs in an anti‐inflammatory manner, targeting the IL‐10, IL‐8, Toll‐like receptor and nuclear factor‐κB signalling pathways. In whole muscle, *IL‐8* mRNA was reduced by 50% and *Jun* mRNA by 25% after exercise training, consistent with the anti‐inflammatory effects of exercise on skeletal muscle. Obesity and 7 days of concurrent exercise training differentially alter skeletal muscle‐derived small EV miRNA contents targeting inflammatory and anabolic pathways.

## 1 INTRODUCTION

Obesity is associated with chronic inflammation (Pedersen & Febbraio, 2012), which is characterized by increased levels of several pro‐inflammatory cytokines, including interleukin 6 (IL‐6), tumor necrosis factor‐α (TNF‐α) and C‐reactive protein (CRP). Chronic inflammation contributes to the development of skeletal muscle insulin resistance, type 2 diabetes mellitus (T2D), cardiovascular disease (CVD) and numerous cancers (Haddad et al., 2005; Lackey & Olefsky, 2016; Pedersen & Febbraio, 2012; Thiebaud et al., 1982). Skeletal muscle is the largest secretory organ in lean humans (Pedersen & Febbraio, 2012) and is responsible for ≤80% of insulin‐stimulated glucose disposal (Thiebaud et al., 1982). Obesity causes biochemical and morphological alterations within skeletal muscle, including insulin resistance (Lackey & Olefsky, 2016; Nicholson et al., 2019), immune cell infiltration (Patsouris et al., 2014) and ectopic fat accumulation (Shulman, 2014), resulting in reduced muscle quality and an increased inflammatory secretome (Collins et al., 2016; Pedersen & Febbraio, 2012).

Myokines are proteins released from skeletal muscle (Febbraio & Pedersen, 2005). Myokines can be regulated by skeletal muscle contraction, act to reduce inflammation and have positive effects on glucose and lipid metabolism (Eckardt et al., 2014; O'Leary et al., 2017). Obesity alters the expression of myokines at rest and after exercise, consistent with increased inflammation and impaired substrate metabolism (Wu & Ballantyne, 2017).

Exercise is a highly effective method for reducing local and systemic inflammation in patients with obesity and T2D, with the greatest reductions coming from concurrent aerobic and resistance exercise training (Balducci et al., 2010; Petersen & Pedersen, 2005; You et al., 2013). Exercise training improves myokine expression in individuals with obesity, resulting in expression more resembling lean control subjects (Shin et al., 2015). Although myokines play a key role in the benefits of exercise training, other factors secreted from skeletal muscle are also important.

Small (30–150 nm) extracellular vesicles (EVs) are secreted by all cells and contain functional mRNAs, microRNAs (miRNAs), lipids and proteins (Thery et al., 2002). miRNAs alter gene expression by binding to target mRNA and act through the RNA silencing complex to reduce target mRNA translation via translational repression or degradation of the target mRNA (Rottiers & Naar, 2012). The impact of small EV miRNAs on cell‐to‐cell signalling is of increasing interest. Adipose tissue‐derived small EVs are a possible regulator of obesity‐associated dysfunction, and small EV signalling might act as an important link between obesity and peripheral tissue insulin sensitivity (Hubal et al., 2017). The miRNA content of small EVs derived from human adipocytes is altered in obese individuals, consistent with observed impairments in peripheral tissue insulin sensitivity (Forterre et al., 2014). In contrast, weight loss alters adipocyte‐derived small EV miRNA, consistent with reduced insulin resistance (Hubal et al., 2017). Small EVs released from lipid‐induced insulin‐resistant muscles modulate gene expression and proliferation of β recipient cells (Jalabert et al., 2016). Small EVs from skeletal muscle improve endothelial cell proliferation, migration and tube formation (Nie et al., 2019). Together, these studies indicate the potential of small EVs to participate in both paracrine and endocrine regulation.

Muscle contraction increases the appearance of small EVs in the systemic venous circulation and the venous circulation draining from muscle (Fruhbeis et al., 2015; Whitham et al., 2018). However, the impact of obesity or exercise training on the miRNA content of human skeletal muscle‐derived small EVs is unknown. We hypothesized that skeletal muscle‐derived small EV miRNA content is altered by: (1) obesity, consistent with greater inflammatory signalling; and (2) 1 week of concurrent exercise training, consistent with reduced inflammation.

## 2 METHODS

### 2.1 Ethical approval

This study conforms to the standards set out by the latest revision of the *Declaration of Helsinki*, except for registration in a database, and was approved by the Purdue University Institutional Review Board (IRB# 1406014975). Eight healthy, sedentary, lean (LN) individuals [three women and five men; body mass index (BMI) < 25 kg/m^2^] and eight healthy, sedentary individuals with obesity (OB; three women and five men; BMI ≥ 30 kg/m^2^) between the ages of 18 and 35 years were recruited to participate in the study. Subjects were non‐smokers with no known chronic disease. Sedentary participants were defined as participating in <1 h of strenuous physical activity per week, and no subject reported any form of regular physical activity. Qualified individuals were administered both verbal and written descriptions of the study. Subjects provided voluntary written consent before the beginning of the study.

### 2.2 Day 1

Subjects reported to the Max E. Wastl Human Performance Laboratory, where height and weight were recorded. Fasting blood was sampled from a cannula inserted into an antecubital vein, for the measurement of insulin, glucose, total cholesterol (TC), high‐density lipoprotein (HDL), low‐density lipoprotein (LDL) and triglycerides (TGs). Additional blood was taken for the measurement of plasma IL‐6, CRP and TNF‐α. The homeostasis model assessment for insulin resistance (HOMA‐IR) and β‐cell function (HOMA‐β) was subsequently calculated (Matthews et al., 1985). After the blood sampling, subjects underwent a vastus lateralis biopsy from a predetermined, randomized leg. Biopsy samples were stored at −80°C until analysis. A section of the biopsy sample (∼100 mg) was placed on ice in EV‐free/serum‐free Dulbecco's modified Eagle's medium for the isolation of skeletal muscle small EVs. Excess muscle was flash frozen and stored at −80°C for further analysis.

### 2.3 Day 2

Subjects reported to the A. H. Ismail Center for Health, Exercise and Nutrition for the determination of maximal oxygen consumption (${\dot{V}}_{O_{2}\max}$) and one‐repetition maximum (1RM). The ${\dot{V}}_{O_{2}\max}$ was measured on an electronically braked cycle ergometer (Excaliber Sport; Lode, Groningen, The Netherlands) as previously described (Gavin et al., 2005). A 5 min warm‐up was performed at 50 W, immediately followed by a 25 W increase every 2 min until volitional fatigue. Minute ventilation (${\dot{V}}_{E}$), oxygen uptake (${\dot{V}}_{O_{2}}$) and carbon dioxide production (${\dot{V}}_{CO_{2}}$) were monitored continuously via open‐circuit spirometry (True Max 2400; Parvo Medics, Salt Lake City, UT, USA). Heart rate (model T31; Polar Electro, Woodbury, NY, USA) and rating of perceived exertion were measured at each workload. Subjects were verbally encouraged to continue for as long as possible. The criteria used to assess ${\dot{V}}_{O_{2}\max}$ included: (1) a heart rate >90% of age‐predicted maximum (220 minus age); (2) a respiratory exchange ratio ≥ 1.10; and (3) identification of a plateau (≤150 ml increase) in ${\dot{V}}_{O_{2}}$ despite a further increase in workload. In all tests, at least two of three criteria were met.

After a 15 min rest period following the ${\dot{V}}_{O_{2}\max}$ test, the leg press (Technogym‐Element, Fairfield, NJ, USA) 1RM was determined. Subjects performed the leg press with their feet shoulder width apart on the platform and with their knees bent at 90° at ∼80% of the subject's body weight. Subjects were given a 30 s to 1 min rest period before attempting the subsequent weight. The weight was increased by 9.1 kg for each consecutive attempt until the subject was unable to extend the knees fully. The highest successfully lifted weight was designated as the 1RM. The 1RM of two lean and six obese subjects exceeded the maximum weight of the equipment (136.4 kg). In these cases, 1RM was estimated based on the maximal number of repetitions the subject was able to complete at 136.4 kg (Brzycki, 1993).

### 2.4 Exercise training protocol

At least 2 days after the initial visit, subjects began a consecutive 7‐day, concurrent exercise training protocol. On each day (days 1–7) subjects performed 45 min of cycle ergometer exercise at 70% ${\dot{V}}_{O_{2}\max}$. In addition on days 2, 4 and 6, subjects performed a bout of resistance exercise consisting of three sets of 8–12 repetitions on the leg press at 80% of 1RM, with 2 min rest between sets.

### 2.5 Final visit

Subjects reported to the Max E. Wastl Human Performance Laboratory 12–14 h after the completion of the exercise training protocol. At this visit, subjects repeated the blood sampling and muscle biopsy procedures.

### 2.6 Blood analysis

Plasma IL‐6, CRP and TNF‐α were measured by Quantikine enzyme‐linked immunosorbent assay (Théry et al., 2018) according to the manufacturer's instructions (R&D Systems, Minneapolis, MN, USA; IL‐6, HS600B; CRP, DCRP00; TNF‐α, HSTA00E). Before analysis, samples were placed on ice and allowed to thaw. All samples were analysed in duplicate, with the average of both values being reported.

### 2.7 Quantitative real‐time PCR

Total muscle RNA was extracted using a TRIzol reagent (Thermo Fisher Scientific) as previously described (Nie et al., 2019). For mRNA reverse transcription, first strand complementary DNA was generated by random hexamer primers with MMLV Reverse Transcriptase (Thermo Fisher Scientific). Real‐time PCR detection was performed using SYBR green‐based chemistry on a CFX Connect (Bio‐Rad, Hercules, CA, USA). Primers for mRNA are listed in the Supporting Information (Table S1). Gene expression was determined with the 2^−ΔΔ^ *^Ct^* relative quantification method and normalized to 18S. Housekeeping genes were validated to ensure that their expression was not influenced by the experimental procedure.

### 2.8 Small extracellular vesicle isolation

After the vastus lateralis biopsies, ∼100 mg of muscle was washed with PBS and gently minced in a 5 cm cell culture dish. Minced muscle was incubated in EV‐free Dulbecco's modified Eagle's medium in standard culture conditions (37°C, air supplemented with 5% CO_2_) for 24 h to facilitate EV secretion. The EV‐containing medium was collected, and small EVs were isolated via differential ultracentrifugation as previously described (Nie et al., 2019). Briefly, medium was centrifuged at 2,000*g* for 10 min, and pelleted cells/debris were discarded. The medium was then centrifuged at 10,000*g* for 30 min at 4°C, followed by filtration through a 0.22 μm syringe filter. Filtered medium was ultracentrifuged at 100,000*g* for 70 min at 4°C. Pelleted EVs were washed with PBS and ultracentrifuged at 100,000*g* for 70 min at 4°C. Small EVs were resuspended in PBS for electron microscopy or in TRIzol reagent (Thermo Fisher Scientific) for the isolation of total EV RNA, as previously described (Nie et al., 2019).

### 2.9 Transmission electron microscopy

**FIGURE 1:**
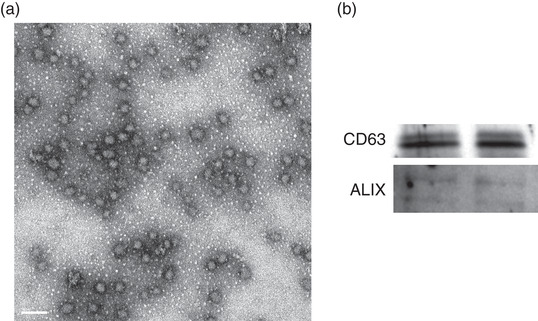
Characterization of skeletal muscle‐derived extracellular vesicles (EVs). (a) Representative transmission electron microscopy image of EVs isolated from whole human skeletal muscle by ultracentrifugation. Scale bar: 100 nm. (b) Representative immunoblot images confirming the presence of the EV markers CD63 and Alix in EVs isolated from whole human muscle

After isolation, the characterization of EVs was performed on a Tecnai T20 transmission electron microscope (FEI, 200 kV) as previously described (Keerthikumar et al., 2015). A representative image of skeletal muscle small EVs is shown in Figure 1. Briefly, small EVs in PBS were pipetted onto carbon‐coated copper electron microscopy grids and incubated for ∼2 min. Excess liquid was blotted away, and grids were washed with water to remove salts. Excess liquid was blotted away, and grids were negatively stained with 2% phosphotungstic acid for 1 min.

### 2.10 Western blot

Western blotting analysis was performed using standard SDS‐PAGE procedures. Briefly, protein was isolated from tissue lysates in RIPA buffer (50 mM Tris–HCl pH 7.4, 150 mM NaCl, 2 mM EDTA, 0.1% SDS, 0.1% Triton X‐100 and 0.5% sodium deoxycholate) with phosphatase inhibitors (0.2 mM NA_3_VO_4_ and 50 mM NaF) and a protease inhibitor cocktail (P8340; Sigma‐Aldrich, St Louis, MO, USA). Next, 20 μg of total protein from small EVs was fractionated on SDS–polyacrylamide gels, transferred to a PVDF membrane and incubated with CD63 (Santa Cruz Biotechnology) and Alix (Cell Signaling Technologies, Danvers, MA, USA) primary antibodies. The membrane was incubated with horseradish peroxide‐conjugated secondary antibodies (Cell Signaling Technology), and images were obtained by chemiluminescence using a ChemiDoc Touch Imaging System and densitometric analysis performed by ImageLab software (Bio‐Rad).

### 2.11 MicroRNA sequencing

Small EV RNA was isolated from a subset of samples (four per group per time point) and analysed by the Purdue University Genomics Core. Raw miRNA sequence reads were analysed by the Purdue University Bioinformatics Core. Adapter trimming followed by clipping of four bases from either end of the reads and quality trimming was performed using Cutadapt software (The Bioo Scientific kit ‘*NEXTflex Illumina Small RNA Sequencing Kit v3*’; version 1.13) (Martin, 2011). After adaptor trimming, reads were trimmed based on quality such that bases above Phred score 30 and reads with a minimum length of five were retained.

Adaptor‐ and quality‐trimmed reads were used by DeconSeq tool (v.0.4.3) (Schmieder & Edwards, 2011) to detect bacterial contamination. DeconSeq tool generates clean and contaminated reads for each sample. Clean reads were extracted using in‐house scripts for downstream analysis. Owing to high levels of bacterial contamination, three samples (two lean and one obese) were excluded from exercise training analysis and one lean sample was excluded from lean versus obese analysis.

Adaptor‐ and quality‐trimmed clean reads were processed through miRDeep2 software (v.2.0.0.8) for miRNA analysis (Friedlander et al., 2012). The preprocessing of reads was achieved through the validated mapper.pl script from miRDeep2, which performs steps such as discarding the reads <15 bp and reads collapsing. Preprocessed reads were mapped against the human reference genome.

Quantification and expression profiling of known miRNAs was performed by the validated quantifier.pl script from miRDeep2 using the collapsed reads and human miRNAs (downloaded from www.miRBase.org). The quantifier script from miRDeep2 generated the read counts for the known miRNAs from each uncontaminated sample. Combined count matrices for all miRNAs and samples were generated using the custom scripts. The DESeq2 package (v.1.16.1) (Love et al., 2014) was used to calculate differential expression of known miRNAs between LN and OB at rest (n = 3 lean/4 obese) and before and after concurrent exercise training (*n* = 5 per time point; two LN and three OB). Owing to a lack of statistical power, we examined the differential response to exercise training between lean and obese qualitatively. We identified the differentially expressed miRNAs of lean and obese following exercise training separately, then cross‐referenced the two lists to generate common and unique differentially expressed miRNAs. The lists of common and unique differentially expressed miRNAs between lean and obese subjects after exercise training, along with fold changes and directionality, are provided in the Supporting Information (Tables S2 and S3).

### 2.12 Biological pathway analysis

Differentially expressed miRNAs between LN and OB (*P* < 0.05; *n* = 22) and between before and after exercise training (*P* < 0.05; *n* = 28) were uploaded into the Ingenuity Pathway Analysis (IPA) suite (Qiagen, Redwood City, CA, USA) for biological pathway analysis. The non‐adjusted *P*‐value was used because the pathway analysis lowers the likelihood of a false positive/negative. IPA uses miRNA seed sequence binding with cognate mRNA targets to identify miRNA–mRNA target interactions across multiple available bioinformatic data sources. We adopted a conservative approach within the miRNA target filter whereby only experimentally verified interactions, determined via miRTarBase (Hsu et al., 2011), and/or highly predicted targets containing binding sites with 8‐mer seed binding, determined via TargetScan (Bartel, 2009), were selected as miRNA–mRNA pairs. This approach yielded a BMI‐related gene target list of mRNAs and an exercise training‐related gene target list of mRNAs. Each gene list was used by IPA to determine enriched canonical pathways targeted by exercise training and obesity miRNAs. Representation of each canonical pathway was tested using Fisher's exact test of ratios of miRNA‐targeted genes in our data set in comparison to the total number of genes in each IPA pathway. The IPA *z*‐score algorithm was used to predict the direction of change for each pathway. A *z*‐score higher than two or less than minus two indicates significant upregulation or downregulation, respectively.

### 2.13 Statistical analysis

An unpaired student's *t*‐test was used to analyse group differences in age, BMI, 1RM and ${\dot{V}}_{O_{2}\max}$. All other data were analysed using a two‐way (two groups × two time points) mixed‐factorial ANOVA. Following a significant *F*‐ratio, Fisher's LSD post‐hoc analysis was performed. Relationships between variables were analysed using linear regression. Significance was established at *P* ≤ 0.05, and data are reported as the mean ± SD. All data were analysed in GraphPad Prism (v.9.20; GraphPad Software, San Diego, CA, USA).

## 3 RESULTS

### 3.1 Subject characteristics

Subject characteristics for all participants are located in Table 1. The subset of subjects for the LN versus OB microRNA sequencing (miRNASEQ) subset are given in Supporting Information Table S4 and for the exercise training subset in Supporting Information Table S5. The characteristics of both the LN versus OB and the exercise training miRNASEQ subset cohorts were representative of the full cohorts, as evidenced by similar trends to those observed in the full cohort. No significant differences were observed between either subset and the full cohort.

**TABLE 1:** Subject characteristics

	**Before exercise training**		**After exercise training**				
**Parameter**	**LN (*n* = 8)**	**OB (*n* = 8)**	**LN (*n* = 8)**	**OB (*n* = 8)**	**INT**	**OB**	**Exercise training**
Age, years	27.0 ± 4.5	26.6 ± 3.3	–	–	–	0.853	–
Height, m	1.8 ± 0.1	1.7 ± 0.1	–	–	–	0.506	–
Weight, kg	74.3 ± 12.9	107.6 ± 21.7	74.3 ± 13.0	106.8 ± 21.7	0.143	0.0019	0.074
BMI, kg/m^2^	22.8 ± 2.3	35.2 ± 5.6	22.9 ± 2.4	35.0 ± 5.7	0.159	0.0001	0.118
Glucose, mg/dl	90.6 ± 10.4	86.3 ± 11.1	89.8 ± 5.5	90.6 ± 9.7	0.347	0.657	0.527
Insulin, μIU/ml	8.8 ± 2.1	29.0 ± 19.5	7.9 ± 2.5	26.4 ± 20.9	0.836	0.0054	0.679
HOMA‐IR, au	1.9 ± 0.4	6.2 ± 4.4	1.7 ± 0.6	5.8 ± 4.5	0.934	0.007	0.763
HOMA‐β, au	138.9 ± 97.0	653.6 ± 546.5	110.4 ± 41.7	395.1 ± 358.7	0.0672	0.0219	0.027
TC, mg/dl	180.3 ± 33.1	190.6 ± 41.8	*163.9 ± 31.1	201.9 ± 36.2	0.0064	–	–
HDL, mg/dl	47.1 ± 9.4	42.4 ± 10.9	47.5 ± 9.2	44.8 ± 15.0	0.481	0.507	0.337
LDL, mg/dl	113.0 ± 29.4	120.9 ± 44.4	101.3 ± 26.5	135 ± 40.1	0.0008	0.259	0.704
TG, mg/dl	100.3 ± 29.0	136.9 ± 41.3	76.4 ± 22.0	110.8 ± 34.8	0.908	0.0175	0.0204
${\dot{V}}_{O_{2}}$_max_, L/min	2.5 ± 0.8	2.8 ± 0.7	–	–	–	0.455	–
${\dot{V}}_{O_{2}}$_max_, ml/kg/min	33.6 ± 5.5	25.6 ± 5.9	–	–	–	0.0142	–
1RM, kg	136.6 ± 49.8	182.6 ± 67.2	–	–	–	0.142	–
1RM, kg/kg	1.8 ± 0.5	1.7 ± 0.5	–	–	–	0.544	–
TNF‐α, pg/μl	0.7 ± 0.3	2.6 ± 5.0	0.6 ± 0.2	0.9 ± 0.2	0.117	0.0372	0.669
CRP, pg/μl	1,175.3 ± 1,134.9	5,283.7 ± 2,477.6	906.2 ± 370.9	5,331.4 ± 2,039.3	0.613	0.0001	0.723
Il‐6, pg/μl	1.4 ± 0.7	3.7 ± 4.6	1.8 ± 2.1	2.0 ± 1.1	0.271	0.171	0.509

Abbreviations: BMI, body mass index; CRP, C‐reactive protein; HDL, high‐density lipoprotein; HOMA‐β, homeostasis model assessment, ‐cell function; HOMA‐IR, homeostasis model assessment, insulin resistance; IL‐6, interleukin‐6; INT, interaction; LDL, low‐density lipoprotein; LN, lean; OB, obese; 1RM, one‐repetition maximum; TC, total cholesterol; TG, triglycerides; TNF‐α, tumor necrosis factor‐α; ${\dot{V}}_{O_{2}}$_max_, maximal oxygen consumption.

*Note*. Values are expressed as the mean ± SD.

^*^Significantly different from all other groups.

As designed, OB had a significantly greater BMI than LN, and there was no observed weight loss in either group as a result of the exercise training. Group OB demonstrated lower relative ${\dot{V}}_{O_{2}\max}$ before training and higher fasting insulin, HOMA‐IR, HOMA‐β, TG, plasma TNF‐α and CRP before and after training. Concurrent exercise training reduced HOMA‐β and TG in both LN and OB groups and reduced TC only in the LN group.

### 3.2 microRNASEQ and IPA analysis by BMI

Differential analysis revealed that 22 miRNAs were differentially expressed between LN and OB (Table 2). Using a conservative filter (only miRNAs with experimentally confirmed or highly conserved predicted targets), we identified that the 22 differentially expressed BMI miRNAs target 3,419 mRNAs (Supporting Information Table S6). Starting with the 3,419 BMI mRNA targets, IPA was used to identify enriched biological pathways. Using a *P*‐value filter of *P* < 0.01, 133 canonical pathways were identified as enriched in the BMI data set (Supporting Information Table S7). Table 3 represents the top 10 (determined by *P*‐value) canonical pathways identified by IPA in the BMI data set. Breaking the top 10 canonical pathways identified broadly into categories, five were related to growth signalling (cardiac hypertrophy, Wnt/β‐catenin, PI3K/AKT, IGF‐1 and PTEN), three to inflammation signalling (PEDF, death receptor and Gα_i_) and two to cancer signalling (molecular mechanisms of cancer and ovarian cancer).

**TABLE 2:** Skeletal muscle extracellular vesicle microRNAs differentially expressed in individuals with obesity in comparison to lean control subjects

	**Raw counts**			
**Mature microRNA**	**Lean**	**Obese**	**Fold change**	***P*‐value**
hsa‐let‐7f‐5p	82,558.7	68,740.5	0.65	0.0321
hsa‐miR‐1‐3p	4,564,095.0	3,056,018.0	0.51	0.0067
hsa‐miR‐1275	3.0	15.8	4.10	0.0080
hsa‐miR‐143‐5p	17.0	63.5	3.27	0.0019
hsa‐miR‐144‐3p	281.3	169.3	0.43	0.0437
hsa‐miR‐155‐5p	26.7	73.5	2.10	0.0050
hsa‐miR‐302b‐3p	0.7	8.5	6.32	0.0265
hsa‐miR‐30c‐1‐3p	48.3	33.5	0.55	0.0293
hsa‐miR‐30e‐5p	39,744.0	27,021.8	0.51	0.0311
hsa‐miR‐3168	13.7	6.8	0.39	0.0293
hsa‐miR‐337‐3p	51.0	114.3	1.75	0.0442
hsa‐miR‐3613‐5p	918.7	580.8	0.47	0.0464
hsa‐miR‐376b‐5p	1.3	9.3	5.58	0.0145
hsa‐miR‐376c‐5p	1.3	9.3	5.58	0.0145
hsa‐miR‐409‐5p	20.7	55.5	2.10	0.0177
hsa‐miR‐432‐5p	18.0	52.8	2.25	0.0193
hsa‐miR‐4485‐3p	0.3	6.8	3.09	0.0279
hsa‐miR‐548a‐3p	11.3	4.5	0.28	0.0195
hsa‐miR‐548ay‐5p	16.7	6.8	0.33	0.0091
hsa‐miR‐654‐5p	2.7	13.5	3.53	0.0177
hsa‐miR‐7641	10.7	36.0	2.80	0.0483
hsa‐miR‐7977	48.7	166.8	2.89	0.0205

**TABLE 3:** Top 10 significant canonical pathways by *P*‐value from biological pathway analysis for differentially expressed skeletal muscle extracellular vesicle microRNAs in individuals with obesity compared with lean control subjects

**Ingenuity canonical pathways**	**−log(*P*‐value)**	**Ratio**	***z*‐score**	**Molecules**
Cardiac hypertrophy signalling (enhanced)	7.4	0.24	−2.01	ACE, ACVR1, ACVR1C, ADRA2A, ADRB2, AGTR1, APEX1, ATF2, ATP2A2, BORCS8‐MEF2B, CAMK2A, CD40LG, CHP1, CTNNB1, DIAPH2, EDN1, EDNRA, EDNRB, EIF2B3, EIF4E, ELK1, ENPP6, FASLG, FGF1, FGF10, FGF16, FGF17, FGF18, FGF20, FGF7, FZD3, FZD6, FZD7, GDPD1, GNA13, GNAI2, GNG2, GNG5, GSK3A, HAND1, HAND2, HDAC4, HDAC7, HSPB2, HSPB7, IGF1, IGF1R, IKBKE, IL13, IL13RA1, IL17C, IL17RD, IL2RB, IL3, IL36G, IL4R, INPP5F, ITGA2, JUN, KRAS, LIF, MAP3K1, MAP3K13, MAP3K2, MAP3K8, MAPK13, MEF2A, MEF2B, MKNK2, MRAS, MYC, NFATC2, NRAS, PDE12, PDE3A, PDE4A, PDE6B, PDE6D, PDE6G, PDE7A, PIK3CB, PIK3R6, PLCD3, PLCH2, PPP3CA, PPP3R1, PPP3R2, PRKACA, PRKACB, PRKAR2A, PRKCA, PRKCG, PRKCI, PTGS2, RALA, RALB, RAP1A, RAP1B, RASD2, RELA, RHOA, ROCK1, RPS6KB1, TGFBR1, TGFBR2, TGFBR3, TNFSF10, TNFSF11, TNFSF13B, TNFSF15, TNFSF9, WNT1, WNT11, WNT4, WNT5A, WNT7B, WNT8A, WNT9A
Molecular mechanisms of cancer	7.24	0.25	–	APAF1, ARHGEF18, ARHGEF3, ATR, BAK1, BCL2, BCL2L1, BMP1, CAMK2A, CASP3, CCND1, CCND2, CCNE2, CDC25A, CDK11B, CDK14, CDK15, CDK18, CDK19, CDK6, CDK8, CDK9, CDKN1A, CDKN1B, CDKN2B, CRK, CTNNB1, CTNND1, CYCS, E2F5, E2F6, E2F7, ELK1, FADD, FANCD2, FAS, FASLG, FOS, FZD3, FZD6, FZD7, GNA13, GNAI2, GNAT1, GRB2, GSK3A, HIF1A, ITGA2, JUN, KRAS, LRP1, MAPK13, MRAS, MYC, NAIP, NFKBIA, NRAS, PAK1, PIK3CB, PIK3R6, PMAIP1, PRKACA, PRKACB, PRKAR2A, PRKCA, PRKCG, PRKCI, PSENEN, RALA, RALB, RAP1A, RAP1B, RASA1, RASD2, RASGRF1, RELA, RHOA, RHOB, RHOG, RHOJ, RND2, SMAD1, SMAD2, SMAD9, SUV39H1, TAB1, TAB2, TCF4, TGFBR1, TGFBR2, TP53, TYK2, WNT1, WNT11, WNT4, WNT5A, WNT7B, WNT8A, WNT9A
Ovarian cancer signalling	5.35	0.30	−0.89	BCL2, CCND1, CD44, CTNNB1, EDN1, EDNRA, EGFR, FSHB, FZD3, FZD6, FZD7, GJA1, KRAS, MLH1, MRAS, NRAS, PIK3CB, PIK3R6, PRKACA, PRKACB, PRKAR2A, PTGS2, RALA, RALB, RAP1A, RAP1B, RASD2, RPS6KB1, SUV39H1, TCF4, TCF7, TCF7L2, TP53, VEGFA, VEGFB, WNT1, WNT11, WNT4, WNT5A, WNT7B, WNT8A, WNT9A
SERPINF1 signalling	5.3	0.35	−0.19	BCL2, BCL2L1, BDNF, ELK1, FAS, FASLG, GDNF, HNF1B, IKBKE, KRAS, MAPK13, MRAS, NFKBIA, NGF, NRAS, PIK3CB, PIK3R6, RALA, RALB, RAP1A, RAP1B, RASD2, RELA, RHOA, ROCK1, TCF4, TCF7, TCF7L2, TP53
Gα_i_ signalling	5.06	0.30	0.69	ADRA2A, AGTR1, APLNR, CHRM2, CNR1, CNR2, DRD3, GNAI2, GNG10, GNG12, GNG13, GNG2, GNG5, GPR17, GRB2, HRH3, HTR1A, HTR1E, HTR1F, KRAS, MRAS, NPR3, NPY1R, NRAS, PRKACA, PRKACB, PRKAR2A, RALA, RALB, RAP1A, RAP1B, RASD2, RGS14, RGS4, RGS7, S1PR1, TBXA2R, XCR1
Wnt/β‐catenin signalling	4.98	0.28	0.60	ACVR1, ACVR1C, CCND1, CD44, CSNK1A1, CSNK1D, CSNK1G2, CSNK2A1, CSNK2A2, CTNNB1, DKK1, DKK4, FZD3, FZD6, FZD7, GJA1, GSK3A, JUN, KREMEN1, LRP1, MAP4K1, MYC, PIN1, PPP2CA, PPP2R1A, PPP2R5A, PTPA, RARB, RARG, SFRP1, SOX12, SOX13, SOX21, TAB1, TCF4, TCF7, TCF7L2, TGFBR1, TGFBR2, TGFBR3, TP53, WNT1, WNT11, WNT4, WNT5A, WNT7B, WNT8A, WNT9A
Death receptor signalling	4.88	0.33	0.37	ACTA1, ACTB, ACTC1, APAF1, BCL2, CASP2, CASP3, CYCS, DFFB, FADD, FAS, FASLG, HSPB2, HSPB7, IKBKE, LIMK1, MAP4K4, NAIP, NFKBIA, PARP11, PARP16, PARP3, RELA, RIPK1, ROCK1, TNFRSF10A, TNFRSF10B, TNFSF10, TNFSF15, TNKS2
PI3K/AKT signalling	4.85	0.30	0.65	BCL2, BCL2L1, CCND1, CDKN1A, CDKN1B, CTNNB1, EIF4E, GRB2, GSK3A, GYS1, IKBKE, INPP5B, INPP5D, INPP5F, INPP5J, ITGA2, KRAS, MAP3K8, MRAS, NFKBIA, NRAS, PIK3CB, PPP2CA, PPP2R1A, PPP2R5A, PTGS2, PTPA, RALA, RALB, RAP1A, RAP1B, RASD2, RELA, RHEB, RPS6KB1, THEM4, TP53, TYK2, YWHAQ
IGF‐1 signalling	4.81	0.31	−1.96	CCN1, CCN2, CCN3, CSNK2A1, CSNK2A2, ELK1, FOS, GRB2, IGF1, IGF1R, IGFBP5, JUN, KRAS, MRAS, NEDD4, NRAS, PIK3CB, PIK3R6, PRKACA, PRKACB, PRKAR2A, PRKCI, RALA, RALB, RAP1A, RAP1B, RASA1, RASD2, RPS6KB1, SOCS1, SOCS3, SOCS4, YWHAQ
PTEN signalling	4.57	0.29	0.17	BCL2, BCL2L1, CASP3, CCND1, CDKN1A, CDKN1B, CSNK2A1, CSNK2A2, EGFR, FASLG, FLT4, FOXG1, FOXO4, GRB2, GSK3A, IGF1R, IKBKE, INPP5B, INPP5D, INPP5F, INPP5J, ITGA2, KRAS, MRAS, NRAS, PIK3CB, RALA, RALB, RAP1A, RAP1B, RASD2, RELA, RPS6KB1, SIRT6, TGFBR1, TGFBR2, TGFBR3

*Note*. The ratio indicates the number of molecules in the data set/total number of molecules in the pathway. The *z*‐score indicates predicted upregulation or downregulation of the pathway compared with lean control subjects.

### 3.3 microRNASEQ and IPA analysis by exercise training

Differential analysis revealed that 28 miRNAs were differentially expressed after exercise training (Table 4). Again, using a conservative filter, we identified that the 28 differentially expressed exercise training miRNAs target 3,070 mRNAs (Supporting Information Table S8). Starting with the 3,070 exercise training mRNA targets, IPA was used to identify enriched biological pathways. Using a *P*‐value filter for pathway significance of *P* < 0.01, 113 canonical pathways were identified as enriched in the exercise training data set (Supporting Information Table S9). Table 5 represents the top 10 canonical pathways identified by IPA in the exercise training data set. Of the top 10 canonical pathways identified, six were related to inflammation signalling (IL‐10, IL‐6, role of macrophages, fibroblasts and endothelial cells in rheumatoid arthritis, Toll‐like receptor, HMGB1and NF‐κB), two to growth signalling (cardiac hypertrophy and Gβγ), one to metabolism (PPAR) and one difficult to identify in muscle signalling (hepatic cholestasis).

**TABLE 4:** Skeletal muscle extracellular vesicle microRNAs differentially expressed after 1 week of concurrent aerobic and resistance exercise training compared with baseline

	**Raw counts**			
**Mature microRNA**	**Pre**	**Post**	**Fold change**	***P*‐value**
hsa‐let‐7f‐2‐3p	87.4	57.0	0.63	0.0382
hsa‐miR‐101‐5p	20.4	9.6	0.47	0.0090
hsa‐miR‐1301‐3p	74.8	132.8	1.91	0.0345
hsa‐miR‐1307‐3p	179.8	317.0	1.81	0.0375
hsa‐miR‐146b‐5p	181.6	312.6	1.87	0.0094
hsa‐miR‐190a‐5p	126.8	71.2	0.53	0.0320
hsa‐miR‐199a‐5p	1,881.2	1,072.2	0.61	0.0462
hsa‐miR‐199b‐5p	2,681.4	1,524.8	0.61	0.0085
hsa‐miR‐208b‐5p	79.2	40.0	0.50	0.0239
hsa‐miR‐23a‐5p	9.4	19.2	2.11	0.0251
hsa‐miR‐296‐3p	3.4	8.0	2.48	0.0302
hsa‐miR‐3605‐3p	4.2	10.0	2.38	0.0355
hsa‐miR‐3609	16.6	8.2	0.49	0.0165
hsa‐miR‐3615	10.6	25.0	2.39	0.0336
hsa‐miR‐370‐3p	33.4	56.8	1.93	0.0124
hsa‐miR‐3960	20.2	43.0	2.39	0.0251
hsa‐miR‐409‐3p	140.6	272.0	1.92	0.0028
hsa‐miR‐4326	2.8	8.4	2.81	0.0282
hsa‐miR‐4485‐3p	18.6	87.2	6.03	0.0002
hsa‐miR‐4485‐5p	3.6	18.4	5.92	0.0009
hsa‐miR‐4488	6.0	31.6	6.36	<0.0001
hsa‐miR‐4497	16.8	35.2	2.34	0.0078
hsa‐miR‐483‐3p	43.6	86.8	1.99	0.0418
hsa‐miR‐483‐5p	22.2	52.6	2.29	0.0196
hsa‐miR‐485‐5p	20.0	37.2	1.90	0.0293
hsa‐miR‐486‐5p	19,897.6	35,386.8	1.82	0.0410
hsa‐miR‐629‐5p	28.0	46.2	1.70	0.0414
hsa‐miR‐7‐5p	141.0	209.6	1.59	0.0383

**TABLE 5:** Top 10 significant canonical pathways by *P*‐value from biological pathway analysis for differentially expressed extracellular vesicle microRNAs after 1 week of concurrent aerobic and resistance exercise training

**Ingenuity canonical pathways**	**−log(*P*‐value)**	**Ratio**	***z*‐score**	**Molecules**
IL‐10 signalling	9.21	0.44	–	CCR5, CHUK, FCGR2A, FOS, HMOX1, IL10, IL10RB, IL1F10, IL1R1, IL1RAP, IL1RAPL2, IL1RL2, IL1RN, IL33, IL36A, IL36B, IL36G, IL36RN, IL37, JUN, LBP, MAP4K4, MAPK13, MAPK14, RELA, SOCS3, SP1, TNF, TRAF6, TYK2
IL‐6 signalling	8.81	0.34	−4.1	AKT2, CHUK, CRP, CSNK2B, CXCL8, FOS, GRB2, HRAS, HSPB7, IL1F10, IL1R1, IL1RAP, IL1RAPL2, IL1RL2, IL1RN, IL33, IL36A, IL36B, IL36G, IL36RN, IL37, IL6ST, JUN, LBP, MAP2K7, MAP4K4, MAPK13, MAPK14, MAPK3, MAPKAPK2, MRAS, NGFR, PIK3R2, RAF1, RAP1A, RASD1, RASD2, RELA, SOCS3, SOS2, TNF, TRAF6, VEGFA
Role of macrophages, fibroblasts and endothelial cells in rheumatoid arthritis	7.83	0.25	–	AKT2, APC2, CAMK2D, CCL5, CEBPA, CHP1, CHUK, CREB1, CREB3L3, CSF1, CXCL8, FOS, FRZB, FZD3, FZD4, FZD6, HRAS, IL10, IL17A, IL1F10, IL1R1, IL1RAP, IL1RAPL2, IL1RL2, IL1RN, IL33, IL36A, IL36B, IL36G, IL36RN, IL37, IL6ST, IRAK1, IRAK2, IRAK4, JUN, LRP1, LRP6, LTB, MAP2K7, MAPK14, MAPK3, MAPKAPK2, MIF, MRAS, NGFR, NOS2, OSM, PDGFC, PIK3R2, PLCH2, PLCZ1, PPP3R2, PRKCQ, PRKCZ, PROK1, RAF1, RAP1A, RASD1, RASD2, RELA, RHOA, ROR2, RYK, SOCS3, TCF7L2, TLR1, TLR10, TLR4, TLR9, TNF, TRAF1, TRAF6, VEGFA, VEGFD, WNT1, WNT2, WNT3A
PPAR signalling	7.68	0.35	4.12	CHUK, CITED2, FOS, GRB2, HRAS, IL1F10, IL1R1, IL1RAP, IL1RAPL2, IL1RL2, IL1RN, IL33, IL36A, IL36B, IL36G, IL36RN, IL37, INS, JUN, MAP4K4, MAPK3, MRAS, NCOR2, NGFR, PDGFC, PDGFRA, PPARA, PPARGC1A, RAF1, RAP1A, RASD1, RASD2, RELA, SOS2, TNF, TRAF6
Toll‐like receptor signalling	7.37	0.38	−3.9	CHUK, FOS, IL12A, IL1F10, IL1RN, IL33, IL36A, IL36B, IL36G, IL36RN, IL37, IRAK1, IRAK2, IRAK4, JUN, LBP, MAP4K4, MAPK13, MAPK14, PPARA, RELA, TLR1, TLR10, TLR4, TLR9, TNF, TOLLIP, TRAF1, TRAF6
Hepatic cholestasis	6.46	0.27	–	ADCY1, CD40LG, CHUK, CXCL8, CYP7B1, IL12A, IL17A, IL17C, IL1F10, IL1R1, IL1RAP, IL1RAPL2, IL1RL2, IL1RN, IL2, IL33, IL36A, IL36B, IL36G, IL36RN, IL37, INS, IRAK1, IRAK2, IRAK4, JUN, LBP, LEP, LTB, NGFR, OSM, PPARA, PRKACA, PRKAG1, PRKAG2, PRKAR1B, PRKCQ, PRKCZ, RARA, RELA, SLC10A1, SLCO1C1, TGFB2, TLR4, TNF, TNFSF10, TNFSF13, TNFSF14, TNFSF4, TRAF6
HMGB1 signalling	6.26	0.28	−3.02	AKT2, CD40LG, CXCL8, FOS, HRAS, IL12A, IL17A, IL17C, IL1F10, IL1R1, IL2, IL33, IL36A, IL36B, IL36G, IL37, JUN, KAT6B, LEP, LTB, MAP2K7, MAPK13, MAPK14, MAPK3, MRAS, NGFR, OSM, PIK3R2, RAC2, RAP1A, RASD1, RASD2, RELA, RHOA, RHOB, RHOG, RHOV, RND1, SP1, TGFB2, TLR4, TNF, TNFSF10, TNFSF13, TNFSF14, TNFSF4
Cardiac hypertrophy signalling (enhanced)	5.66	0.21	−4.75	ADCY1, AKT2, ATP2A3, CAMK2D, CD40LG, CHP1, CHUK, CTF1, CXCL8, EDN1, EDNRA, EIF2B5, EIF4E, FGF1, FGF10, FGF11, FGF14, FGF17, FGF23, FGF7, FICD, FZD3, FZD4, FZD6, GDE1, GDPD1, GNA11, GNAI2, GNG5, GSK3A, H2BFM, HDAC4, HDAC8, HRAS, HSPB7, IFNAR1, IGF1, IL10RB, IL12A, IL12RB2, IL17A, IL17C, IL1F10, IL1R1, IL1RL2, IL2, IL2RB, IL33, IL36A, IL36B, IL36G, IL37, IL6ST, ITGA5, JUN, LEP, LTB, MAP2K7, MAP3K11, MAP3K8, MAP3K9, MAPK13, MAPK14, MAPK3, MAPKAPK2, MKNK1, MKNK2, MRAS, NGFR, NKX2‐5, NPPA, OSM, PDE2A, PDE4A, PIK3R2, PLCH2, PLCZ1, PLN, PPP3R2, PRKACA, PRKAG1, PRKAG2, PRKAR1B, PRKCQ, PRKCZ, PTEN, RAF1, RAP1A, RASD1, RASD2, RELA, RHOA, TGFB2, TNF, TNFSF10, TNFSF13, TNFSF14, TNFSF4, WNT1, WNT2, WNT3A
Gβγ signalling	5.29	0.29	−4.35	ADCY1, AKT2, BTK, CACNA1B, CACNA2D4, CACNB1, CACNB2, CACNB4, CACNG2, CACNG4, CACNG7, CACNG8, CAV3, EGFR, GNA11, GNAI2, GNAT1, GNG5, GRB2, HRAS, KCNJ5, MAPK3, MRAS, PAK1, PRKACA, PRKAG1, PRKAG2, PRKAR1B, PRKCQ, PRKCZ, RAF1, RAP1A, RASD1, RASD2, SOS2
Nuclear factor‐κB signalling	5.21	0.26	−5.73	AKT2, BCL10, CD40, CD40LG, CHUK, CSNK2B, EGFR, FADD, HRAS, IL1F10, IL1R1, IL1RN, IL33, IL36A, IL36B, IL36G, IL36RN, IL37, INS, IRAK1, IRAK4, MAP2K7, MAP3K8, MAP4K4, MRAS, NGFR, PDGFRA, PIK3R2, PRKACA, PRKCQ, PRKCZ, RAF1, RAP1A, RASD1, RASD2, RELA, TGFA, TLR1, TLR10, TLR4, TLR9, TNF, TNFRSF11A, TNIP1, TRAF6, ZAP70

*Note*. The ratio indicates the number of molecules in the data set/total number of molecules in the pathway. The *z*‐score indicates predicted upregulation or downregulation of the pathway compared with lean control subjects.

### 3.4 Muscle mRNA

**FIGURE 2:**
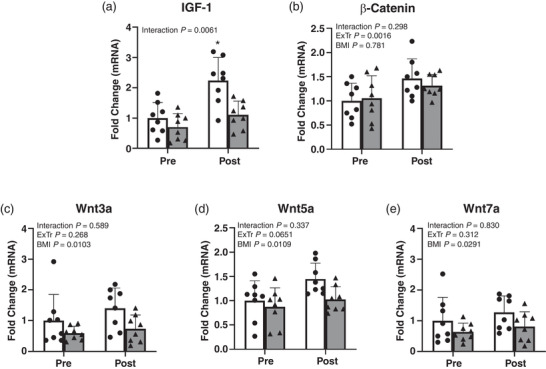
Whole skeletal muscle mRNA expression for (a) *IGF‐1*, (b) β‐catenin, (c) *Wnt3a*, (d) *Wnt5a* and (e) *Wnt7a* before and after exercise training in lean (LN) and obese (OB) humans. ^*^Significantly different from all other groups (*P* ≤ 0.05). LN Pre was set to one. Black bars, LN; grey bars, OB. Values are the mean ± SD; *n *= 8 per group

Whole skeletal muscle mRNAs for components of the Wnt/β‐catenin and IGF‐1 signalling pathways were measured to investigate the results from pathway analysis further (Figure 2). A significant interaction effect was observed for *IGF‐1* mRNA, whereby *IGF‐1* mRNA was increased in LN after exercise training compared with LN before and OB before and after exercise training. The expression of β‐catenin mRNA was increased by 50% after exercise training, but no differences were observed between LN and OB. The expression of key Wnt ligands, *Wnt3a*, *Wnt5a* and *Wnt7a*, were all reduced in OB at rest and after concurrent exercise training by approximately 45, 20 and 35%, respectively. A trend was observed for an increase in *Wnt5a* expression after concurrent exercise training (*P* = 0.07).

**FIGURE 3:**
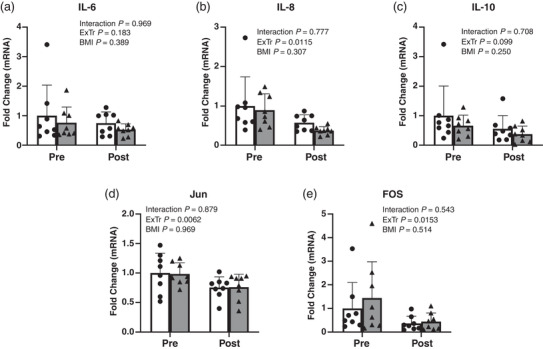
Whole skeletal muscle mRNA expression for (a) *IL‐6*, (b) *IL‐8*, (c) *IL‐10*, (d) *Jun* and (e) *FOS* before and after exercise training in lean (LN) and obese (OB) humans. LN Pre was set to one. Black bars, LN; grey bars, OB. Values are the mean ± SD; *n *= 8 per group

Whole skeletal muscle mRNAs for components of the IL‐6 and IL‐10 pathways are shown in Figure 3. One week of concurrent exercise training reduced *IL‐8*, *Jun* and *FOS* mRNA expression in both LN and OB by approximately 50, 25 and 65%, respectively. A trend for a reduction in *IL‐10* was observed after exercise training (*P* = 0.10). No differences were observed in muscle expression of *IL‐6*, *IL‐8*, *IL‐10*, *Jun* or *FOS* mRNA between LN and OB either before or after exercise training.

## 4 DISCUSSION

The present study demonstrates that obesity alters skeletal muscle‐derived small EV miRNAs targeting many inflammatory and anabolic pathways, including the Wnt/β‐catenin and IGF‐1 signalling pathways. In addition, 1 week of concurrent aerobic and resistance exercise training alters skeletal muscle‐derived small EV miRNAs targeting several inflammatory pathways, including the IL‐6 and IL‐10 pathways, indicative of reduced inflammation. To our knowledge, this is the first report demonstrating that obesity and exercise training alter the miRNA content of skeletal muscle‐derived small EVs.

### 4.1 Obesity alters EV miRNAs targeting anabolic pathways

Obesity is a disease of chronic inflammation characterized by an increase in circulating inflammatory cytokines (Haddad et al., 2005; Pedersen & Febbraio, 2012). Confirming this, circulating TNF‐α and CRP were higher in individuals with than without obesity (Table 1). We hypothesized that small EVs isolated from skeletal muscle of individuals with obesity would contain pro‐inflammatory signals. Consistent with this hypothesis, three signalling pathways identified were related to inflammation: SERPINF1, death receptor and Gα_i_.

However, the larger share of pathways (5 of 10) were related to growth: cardiac hypertrophy, Wnt/β‐catenin, PI3K/AKT, IGF‐1 and PTEN. Biological pathway analysis found that obesity alters the expression of small EV miRNAs targeting components of the IGF‐1 and Wnt/β‐catenin signalling pathways. In addition to growth signalling, the Wnt/β‐catenin signalling pathway regulates the release of pro‐ and anti‐inflammatory cytokines in different cell types and is implicated in the progression of metabolic syndrome (Ma & Hottiger, 2016). Alterations in Wnt/β‐catenin signalling could contribute to an increase in the production of pro‐inflammatory cytokines, such as IL‐6, IL‐8 and TNF‐α (Ma & Hottiger, 2016).

Individuals with obesity appear to be resistant to anabolic stimuli, which is likely to be the result of insulin resistance, lipid accumulation and/or inflammation (Beals et al., 2019). Anabolic resistance in obese individuals can lead to a reduction in the regenerative capacity of skeletal muscle after injury (Fu et al., 2016). Impairments in IGF‐1/AKT/mTOR and Wnt/β‐catenin signalling have been observed in obese animals during muscle regeneration, suggesting that impaired signalling in these pathways has negative consequences for the maintenance of skeletal muscle mass (Brown et al., 2015; Zhou et al., 2012). Recently, we reported that skeletal muscle *IGF‐1* mRNA and protein and the mRNA expression of key Wnt ligands are reduced at rest and after acute resistance exercise in humans with obesity (Sullivan et al., 2020). In the present study, skeletal muscle *IGF‐1*, *Wnt3a*, *Wnt5a* and *Wnt7a* mRNAs were all reduced in subjects with obesity. Skeletal muscle EVs regulate Wnt signalling during myogenesis (Forterre et al., 2014; Rome et al., 2019), indicating that altered small EV content could contribute to impaired regenerative capacity in obesity (Fu et al., 2016). Consistent with a role for small EVs in cell‐to‐cell communication, the present results suggest that muscle intracellular growth and inflammation status are communicated via small EVs.

### 4.2 Exercise training alters EV miRNAs targeting inflammation

Concurrent aerobic and resistance exercise training has potent anti‐inflammatory effects and reduces local and systemic inflammation in individuals with or without obesity (Balducci et al., 2010; Petersen & Pedersen, 2005; You et al., 2013). In the present report, 1 week of concurrent aerobic and resistance exercise training did not reduce the circulating markers of inflammation (IL‐6, TNF‐α or CRP) in either LN or OB individuals. This is likely to be attributable to the short duration of exercise training in the present study, suggesting that a longer duration of exercise training is necessary to observe changes in these circulating inflammatory markers.

One week of concurrent aerobic and resistance exercise training did reduce markers of intracellular muscle inflammation, because the gene expressions of *IL‐8*, *Jun* and *FOS* were reduced after exercise training. Consistent with intercellular communication of intracellular muscle status, the majority of pathways (6 of 10) targeted by muscle small EV miRNAs and altered by concurrent exercise training were related to inflammation signalling: IL‐10, IL‐6, role of macrophages, fibroblasts and endothelial cells in rheumatoid arthritis, Toll‐like receptor, HMGB1 and NF‐κB.

Additionally, small EV miRNAs were altered after concurrent aerobic and resistance exercise training, consistent with increased PPAR signalling. Upregulation of PPAR signalling leads to improvements in exercise tolerance, lipid metabolism and mitochondrial biogenesis via peroxisome proliferator‐activated receptor gamma coactivator 1‐alpha (PGC1‐α) (Calvo et al., 2008). Increased mitochondrial biogenesis is a hallmark of adaptation to aerobic exercise training. Although our knowledge of the regulation of muscle small EV content and release remains limited, it would be anticipated that changes in intracellular status with chronic disease or exercise training (anti‐inflammation, mitochondrial biogenesis, etc.) would translate to alterations in small EV intercellular communication.

The target cells for the small EVs released from skeletal muscle are an important, but poorly explored topic. In mice, muscle‐derived EVs are present in the circulation, triple in response to exercise and contribute to the systemic antioxidant defense (Gao et al., 2021). Recently, we have demonstrated the potent paracrine effects that small skeletal muscle‐derived EVs are capable of exerting on endothelial cells. Small muscle‐derived EVs improve inflammatory signalling and increase endothelial cell proliferation, migration and tube formation via activation of the nuclear factor‐κB pathway (Nie et al., 2019). Recent evidence indicates that EVs exert endocrine effects in addition to their potent paracrine effects (Rome et al., 2019). However, the extent of these endocrine effects is likely to be limited, because only 5% of circulating EVs are of myogenic origin despite the robust ability of skeletal muscle to secrete EVs, (Estrada et al., 2021). After intraperitoneal injection in mice, skeletal muscle‐derived EVs can be found within cells of at least eight different organs in addition to skeletal muscle, including brain, liver, heart, lungs, gastrointestinal tract, spleen, kidney and pancreas (Jalabert et al., 2016). Additionally, it appears that skeletal muscle EVs contribute to the crossover effects of unilateral exercise (Pietrangelo et al., 2018). Twenty‐four hours after injection of green fluorescent protein‐labelled skeletal muscle EVs into the right tibialis anterior of mice, fluorescence was detected in the right quadriceps and the left tibialis anterior (Jalabert et al., 2016). Thus, small skeletal muscle‐derived EVs have both paracrine and endocrine functions, and this raises the possibility that they might contribute to exercise‐induced improvements in systemic inflammation.

### 4.3 Limitations

Skeletal muscle contains a variety of cell types, including endothelial cells, satellite cells, neural cells, macrophages and pericytes. Thus, it is possible that the EVs isolated were not from myofibres. However, the contribution of EVs of non‐skeletal muscle origin is likely to be minimal, because ∼80% of total mapped reads were for skeletal muscle‐specific miRNAs (miR‐1, miR‐133, miR‐206, miR‐486 and miR‐499; data not shown).

### 4.4 Conclusion

In conclusion, obesity alters the miRNA content of small skeletal muscle‐derived EVs targeting several growth and inflammatory pathways, including the anabolic pathways IGF‐1 and Wnt/β‐catenin. Also, 1 week of concurrent aerobic and resistance exercise training alters the miRNA content of small skeletal muscle‐derived EVs targeting several inflammatory pathways, including IL‐6 and IL‐10, indicative of reduced inflammation. Thus, skeletal muscle EV content is different in individuals with obesity compared with lean individuals, but exercise training is able to alter miRNAs towards a beneficial signalling profile. Considerable work remains in understanding the physiological role of small muscle‐derived EVs in cell‐to‐cell communication in health and disease.

## COMPETING INTERESTS

None declared.

## AUTHOR CONTRIBUTIONS

B.P.S., Y.N., S.K., J.S. and T.P.G. contributed to the conception and design of the work. B.P.S., Y.N., S.E., C.K.K., Z.R.H., R.T.G., M.J.H. and T.P.G. contributed to the acquisition, analysis or interpretation of data for the work. B.P.S. and T.P.G. drafted the manuscript. All authors critically revised the manuscript for important intellectual content, approved the final version of the manuscript and agree to be accountable for all aspects of the work in ensuring that questions related to the accuracy or the integrity of any part of the work are appropriately investigated and resolved. All persons designated as authors qualify for authorship, and all those who qualify for authorship are listed.

## FUNDING INFORMATION

This research project was supported by intramural funds from Purdue University. T.P.G. was supported, in part, during the preparation of this manuscript by the American Heart Association (grant 20IPA35360013).

## Supporting information

Statistical Summary Document

**Table** **S1**. Primer sequences for qRT‐PCR.
**Table S2**. Common differentially expressed skeletal muscle extracellular vesicle miRNAs between individuals with obesity and lean controls following one week of concurrent exercise training. Fold change < 0.8 = down. Fold change > 1.2 = up. ‐ = no change.
**Table S3**. Uncommon differentially expressed skeletal muscle extracellular vesicle miRNAs between individuals with obesity and lean controls following one week of concurrent exercise training. Fold change < 0.8 = down. Fold change > 1.2 = up. ‐ = no change.
Table S4. Subject characteristics for lean (LN) vs obese (OB) miRNA SEQ samples. V̇O2MAX ‐ maximal oxygen consumption; 1 RM‐ one repetition maximum; BMI ‐ body mass index; HOMA‐IR ‐ homeostasis model assessment – insulin resistance; HOMA‐β ‐ homeostasis model assessment – β‐cell function; TC – Total Cholesterol; HDL ‐ high density lipoprotein; LDL ‐ low density lipoprotein; TG – Triglycerides; TNF‐α ‐ Tumor Necrosis Factor α; CRP ‐ C‐reactive Protein; IL‐6 ‐ Interleukin‐6. Mean ± SE.
Table S5. Subject characteristics for exercise training miRNA SEQ samples. V̇O2MAX ‐ maximal oxygen consumption; 1 RM‐ one repetition maximum; BMI ‐ body mass index; HOMA‐IR ‐ homeostasis model assessment – insulin resistance; HOMA‐β ‐ homeostasis model assessment – β‐cell function; TC – Total Cholesterol; HDL ‐ high density lipoprotein; LDL ‐ low density lipoprotein; TG – Triglycerides; TNF‐α ‐ Tumor Necrosis Factor α; CRP ‐ C‐reactive Protein; IL‐6 ‐ Interleukin‐6. Mean ± SE.

Table S6. mRNA targets of differentially expressed microRNA between lean and obese.

Table S7. Significant canonical pathways identified by pathway analysis for differentially expressed extracellular vesicle microRNA between lean and obese.

Table S8. mRNA targets for differentially expresed microRNAs after exercise training.

Table S9. Significant canonical pathways identified by pathway analysis for differentially expressed extracellular vesicle microRNA after exercise training.

EV miRNA Raw Counts

Whole Muscle mRNA

## Data Availability

The data that support the findings of this study are available in the Supporting Information for this article or from the corresponding author upon reasonable request.

## References

[eph13161-bib-0001] Balducci, S. , Zanuso, S. , Nicolucci, A. , Fernando, F. , Cavallo, S. , Cardelli, P. , Fallucca, S. , Alessi, E. , Letizia, C. , Jimenez, A. , Fallucca, F. , & Pugliese, G. (2010). Anti‐inflammatory effect of exercise training in subjects with type 2 diabetes and the metabolic syndrome is dependent on exercise modalities and independent of weight loss. Nutrition, Metabolism and Cardiovascular Diseases, 20(8), 608–617. 10.1016/j.numecd.2009.04.015 19695853

[eph13161-bib-0002] Bartel, D. P. (2009). MicroRNAs: Target recognition and regulatory functions. Cell, 136(2), 215–233. 10.1016/j.cell.2009.01.002 19167326 PMC3794896

[eph13161-bib-0003] Beals, J. W. , Burd, N. A. , Moore, D. R. , & Van Vliet, S. (2019). Obesity alters the muscle protein synthetic response to nutrition and exercise. Frontiers in Nutrition, 6, 87. 10.3389/fnut.2019.00087 31263701 PMC6584965

[eph13161-bib-0004] Brown, L. A. , Lee, D. E. , Patton, J. F. , Perry, R. A. , Brown, J. L. , Baum, J. I. , Smith‐Blair, N. , Greene, N. P. , & Washington, T. A. (2015). Diet‐induced obesity alters anabolic signalling in mice at the onset of skeletal muscle regeneration. Acta Physiologica (Oxford, England), 215(1), 46–57. 10.1111/apha.12537 26052759

[eph13161-bib-0005] Brzycki, M. (1993). Strength testing—Predicting a one‐rep max from reps‐to‐fatigue. Journal of Physical Education, Recreation & Dance, 64(1), 88–90. 10.1080/07303084.1993.10606684

[eph13161-bib-0006] Calvo, J. A. , Daniels, T. G. , Wang, X. , Paul, A. , Lin, J. , Spiegelman, B. M. , Stevenson, S. C. , & Rangwala, S. M. (2008). Muscle‐specific expression of PPARγ coactivator‐1α improves exercise performance and increases peak oxygen uptake. Journal of Applied Physiology, 104(5), 1304–1312. 10.1152/japplphysiol.01231.2007 18239076

[eph13161-bib-0007] Collins, K. H. , Paul, H. A. , Hart, D. A. , Reimer, R. A. , Smith, I. C. , Rios, J. L. , Seerattan, R. A. , & Herzog, W. (2016). A high‐fat high‐sucrose diet rapidly alters muscle integrity, inflammation and gut microbiota in male rats. Science Reports, 6, 37278. 10.1038/srep37278 PMC5112513 27853291

[eph13161-bib-0008] Eckardt, K. , Görgens, S. W. , Raschke, S. , & Eckel, J. (2014). Myokines in insulin resistance and type 2 diabetes. Diabetologia, 57(6), 1087–1099. 10.1007/s00125-014-3224-x 24676645

[eph13161-bib-0009] Estrada, A. L. , Valenti, Z. J. , Hehn, G. , Amorese, A. J. , Williams, N. S. , Balestrieri, N. P. , Deighan, C. , Allen, C. P. , Spangenburg, E. E. , Kruh‐Garcia, N. A. , & Lark, D. S. (2021). Extracellular vesicle secretion is tissue‐dependent ex vivo and skeletal muscle myofiber extracellular vesicles reach the circulation in vivo. American Journal of Physiology. Cell Physiology, 322(2), C246–C259. 10.1152/ajpcell.00580.2020 34910603 PMC8816621

[eph13161-bib-0010] Febbraio, M. A. , & Pedersen, B. K. (2005). Contraction‐induced myokine production and release: Is skeletal muscle an endocrine organ? Exercise and Sport Sciences Reviews, 33(3), 114–119. 10.1097/00003677-200507000-00003 16006818

[eph13161-bib-0011] Forterre, A. , Jalabert, A. , Berger, E. , Baudet, M. , Chikh, K. , Errazuriz, E. , De Larichaudy, J. , Chanon, S. , Weiss‐Gayet, M. , Hesse, A.‐M. , Record, M. , Geloen, A. , Lefai, E. , Vidal, H. , Couté, Y. , & Rome, S. (2014). Proteomic analysis of C2C12 myoblast and myotube exosome‐like vesicles: A new paradigm for myoblast‐myotube cross talk? PLoS One, 9(1), e84153. 10.1371/journal.pone.0084153 24392111 PMC3879278

[eph13161-bib-0012] Friedländer, M. R. , Mackowiak, S. D. , Li, N. , Chen, W. , & Rajewsky, N. (2012). miRDeep2 accurately identifies known and hundreds of novel microRNA genes in seven animal clades. Nucleic Acids Research, 40(1), 37–52. 10.1093/nar/gkr688 21911355 PMC3245920

[eph13161-bib-0013] Frühbeis, C. , Helmig, S. , Tug, S. , Simon, P. , & Krämer‐Albers, E.‐M. (2015). Physical exercise induces rapid release of small extracellular vesicles into the circulation. Journal of Extracellular Vesicles, 4, 28239. 10.3402/jev.v4.28239 26142461 PMC4491306

[eph13161-bib-0014] Fu, X. , Zhu, M. , Zhang, S. , Foretz, M. , Viollet, B. , & Du, M. (2016). Obesity impairs skeletal muscle regeneration through inhibition of AMPK. Diabetes, 65(1), 188–200. 10.2337/db15-0647 26384382 PMC4686944

[eph13161-bib-0015] Gao, L. , Wang, H.‐J. , Tian, C. , & Zucker, I. H. (2021). Skeletal muscle Nrf2 contributes to exercise‐evoked systemic antioxidant defense via extracellular vesicular communication. Exercise and Sport Sciences Reviews, 49(3), 213–222. 10.1249/JES.0000000000000257 33927165 PMC8195856

[eph13161-bib-0016] Gavin, T. P. , Stallings, H. W. , Zwetsloot, K. A. , Westerkamp, L. M. , Ryan, N. A. , Moore, R. A. , Pofahl, W. E. , & Hickner, R. C. (2005). Lower capillary density but no difference in VEGF expression in obese vs. lean young skeletal muscle in humans. Journal of Applied Physiology, 98(1), 315–321. 10.1152/japplphysiol.00353.2004 15298982

[eph13161-bib-0017] Haddad, F. , Zaldivar, F. , Cooper, D. M. , & Adams, G. R. (2005). IL‐6‐induced skeletal muscle atrophy. Journal of Applied Physiology, 98(3), 911–917. 10.1152/japplphysiol.01026.2004 15542570

[eph13161-bib-0018] Hsu, S.‐D. , Lin, F.‐M. , Wu, W.‐Y. , Liang, C. , Huang, W.‐C. , Chan, W.‐L. , Tsai, W.‐T. , Chen, G.‐Z. , Lee, C.‐J. , Chiu, C.‐M. , Chien, C.‐H. , Wu, M.‐C. , Huang, C.‐Y. , Tsou, A.‐P. , & Huang, H.‐D. (2011). miRTarBase: A database curates experimentally validated microRNA‐target interactions. Nucleic Acids Research, 39(Database issue), D163–D169. 10.1093/nar/gkq1107 21071411 PMC3013699

[eph13161-bib-0019] Hubal, M. J. , Nadler, E. P. , Ferrante, S. C. , Barberio, M. D. , Suh, J.‐H. , Wang, J. , Dohm, G. L. , Pories, W. J. , Mietus‐Snyder, M. , & Freishtat, R. J. (2017). Circulating adipocyte‐derived exosomal MicroRNAs associated with decreased insulin resistance after gastric bypass. Obesity (Silver Spring), 25(1), 102–110. 10.1002/oby.21709 27883272 PMC5182153

[eph13161-bib-0020] Jalabert, A. , Vial, G. , Guay, C. , Wiklander, O. P. B. , Nordin, J. Z. , Aswad, H. , Forterre, A. , Meugnier, E. , Pesenti, S. , Regazzi, R. , Danty‐Berger, E. , Ducreux, S. , Vidal, H. , El‐Andaloussi, S. , Rieusset, J. , & Rome, S. (2016). Exosome‐like vesicles released from lipid‐induced insulin‐resistant muscles modulate gene expression and proliferation of beta recipient cells in mice. Diabetologia, 59(5), 1049–1058. 10.1007/s00125-016-3882-y 26852333

[eph13161-bib-0021] Keerthikumar, S. , Gangoda, L. , Liem, M. , Fonseka, P. , Atukorala, I. , Ozcitti, C. , Mechler, A. , Adda, C. G. , Ang, C.‐S. , & Mathivanan, S. (2015). Proteogenomic analysis reveals exosomes are more oncogenic than ectosomes. Oncotarget, 6(17), 15375–15396. 10.18632/oncotarget.3801 25944692 PMC4558158

[eph13161-bib-0022] Lackey, D. E. , & Olefsky, J. M. (2016). Regulation of metabolism by the innate immune system. Nature Reviews Endocrinology, 12(1), 15–28. 10.1038/nrendo.2015.189 26553134

[eph13161-bib-0023] Love, M. I. , Huber, W. , & Anders, S. (2014). Moderated estimation of fold change and dispersion for RNA‐seq data with DESeq2. Genome Biology, 15(12), 550. 10.1186/s13059-014-0550-8 25516281 PMC4302049

[eph13161-bib-0024] Ma, B. , & Hottiger, M. O. (2016). Crosstalk between Wnt/β‐catenin and NF‐κB signaling pathway during inflammation. Frontiers in Immunology, 7, 378. 10.3389/fimmu.2016.00378 27713747 PMC5031610

[eph13161-bib-0025] Martin, M. (2011). Cutadapt removes adapter sequences from high‐throughput sequencing reads. EMBnet.journal, 17(1), 10. 10.14806/ej.17.1.200

[eph13161-bib-0026] Matthews, D. R. , Hosker, J. P. , Rudenski, A. S. , Naylor, B. A. , Treacher, D. F. , & Turner, R. C. (1985). Homeostasis model assessment: Insulin resistance and beta‐cell function from fasting plasma glucose and insulin concentrations in man. Diabetologia, 28(7), 412–419. 10.1007/BF00280883 3899825

[eph13161-bib-0027] Nicholson, T. , Church, C. , Tsintzas, K. , Jones, R. , Breen, L. , Davis, E. T. , Baker, D. J. , & Jones, S. W. (2019). Vaspin promotes insulin sensitivity of elderly muscle and is upregulated in obesity. Journal of Endocrinology, 241(1), 31–43. 10.1530/JOE-18-0528 30721136

[eph13161-bib-0028] Nie, Y. , Sato, Y. , Garner, R. T. , Kargl, C. , Wang, C. , Kuang, S. , Gilpin, C. J. , & Gavin, T. P. (2019). Skeletal muscle‐derived exosomes regulate endothelial cell functions via reactive oxygen species‐activated nuclear factor‐κB signalling. Experimental Physiology, 104(8), 1262–1273. 10.1113/EP087396 31115069

[eph13161-bib-0029] O'Leary, M. F. , Wallace, G. R. , Bennett, A. J. , Tsintzas, K. , & Jones, S. W. (2017). IL‐15 promotes human myogenesis and mitigates the detrimental effects of TNFα on myotube development. Scientific Reports, 7(1), 12997. 10.1038/s41598-017-13479-w 29021612 PMC5636823

[eph13161-bib-0030] Patsouris, D. , Cao, J.‐J. , Vial, G. , Bravard, A. , Lefai, E. , Durand, A. , Durand, C. , Chauvin, M.‐A. , Laugerette, F. , Debard, C. , Michalski, M.‐C. , Laville, M. , Vidal, H. , & Rieusset, J. (2014). Insulin resistance is associated with MCP1‐mediated macrophage accumulation in skeletal muscle in mice and humans. PLoS One, 9(10), e110653. 10.1371/journal.pone.0110653 25337938 PMC4206428

[eph13161-bib-0031] Pedersen, B. K. , & Febbraio, M. A. (2012). Muscles, exercise and obesity: Skeletal muscle as a secretory organ. Nature Reviews Endocrinology, 8(8), 457–465. 10.1038/nrendo.2012.49 22473333

[eph13161-bib-0032] Petersen, A. M. W. , & Pedersen, B. K. (2005). The anti‐inflammatory effect of exercise. Journal of Applied Physiology, 98(4), 1154–1162. 10.1152/japplphysiol.00164.2004 15772055

[eph13161-bib-0033] Pietrangelo, T. , Bondi, D. , Kinel, E. , & Verratti, V. (2018). The bottom‐up rise strength transfer in elderly after endurance and resistance training: The BURST. Frontiers in Physiology, 9, 1944. 10.3389/fphys.2018.01944 30692938 PMC6339983

[eph13161-bib-0034] Rome, S. , Forterre, A. , Mizgier, M. L. , & Bouzakri, K. (2019). Skeletal muscle‐released extracellular vesicles: State of the art. Frontiers in Physiology, 10, 929. 10.3389/fphys.2019.00929 31447684 PMC6695556

[eph13161-bib-0035] Rottiers, V. , & Näär, A. M. (2012). MicroRNAs in metabolism and metabolic disorders. Nature Reviews Molecular Cell Biology, 13(4), 239–250. 10.1038/nrm3313 22436747 PMC4021399

[eph13161-bib-0036] Schmieder, R. , & Edwards, R. (2011). Fast identification and removal of sequence contamination from genomic and metagenomic datasets. PLoS One, 6(3), e17288. 10.1371/journal.pone.0017288 21408061 PMC3052304

[eph13161-bib-0037] Shin, K. O. , Bae, J. Y. , Woo, J. , Jang, K. S. , Kim, K. S. , Park, J. S. , Kim, I. K. , & Kang, S. (2015). The effect of exercise on expression of myokine and angiogenesis mRNA in skeletal muscle of high fat diet induced obese rat. Journal of Exercise Nutrition & Biochemistry, 19(2), 91–98. 10.5717/jenb.2015.15061006 26244127 PMC4523810

[eph13161-bib-0038] Shulman, G. I. (2014). Ectopic fat in insulin resistance, dyslipidemia, and cardiometabolic disease. New England Journal of Medicine, 371(12), 1131–1141. 10.1056/NEJMra1011035 25229917

[eph13161-bib-0039] Sullivan, B. P. , Weiss, J. A. , Nie, Y. , Garner, R. T. , Drohan, C. J. , Kuang, S. , Stout, J. , & Gavin, T. P. (2020). Skeletal muscle IGF‐1 is lower at rest and after resistance exercise in humans with obesity. European Journal of Applied Physiology, 120, 2835–2846. 10.1007/s00421-020-04509-z 32989478

[eph13161-bib-0040] Théry, C. , Witwer, K. W. , Aikawa, E. , Alcaraz, M. J. , Anderson, J. D. , Andriantsitohaina, R. , Antoniou, A. , Arab, T. , Archer, F. , Atkin‐Smith, G. K. , Ayre, D. C. , Bach, J.‐M. , Bachurski, D. , Baharvand, H. , Balaj, L. , Baldacchino, S. , Bauer, N. N. , Baxter, A. A. , Bebawy, M. , … Zuba‐Surma, E. K. (2018). Minimal information for studies of extracellular vesicles 2018 (MISEV2018): A position statement of the International Society for Extracellular Vesicles and update of the MISEV2014 guidelines. Journal of Extracellular Vesicles, 7(1), 1535750. 10.1080/20013078.2018.1535750 30637094 PMC6322352

[eph13161-bib-0041] Théry, C. , Zitvogel, L. , & Amigorena, S. (2002). Exosomes: Composition, biogenesis and function. Nature Reviews Immunology, 2(8), 569–579. 10.1038/nri855 12154376

[eph13161-bib-0042] Thiebaud, D. , Jacot, E. , Defronzo, R. A. , Maeder, E. , Jequier, E. , & Felber, J.‐P. (1982). The effect of graded doses of insulin on total glucose uptake, glucose oxidation, and glucose storage in man. Diabetes, 31(11), 957–963. 10.2337/diacare.31.11.957 6757014

[eph13161-bib-0043] Whitham, M. , Parker, B. L. , Friedrichsen, M. , Hingst, J. R. , Hjorth, M. , Hughes, W. E. , Egan, C. L. , Cron, L. , Watt, K. I. , Kuchel, R. P. , Jayasooriah, N. , Estevez, E. , Petzold, T. , Suter, C. M. , Gregorevic, P. , Kiens, B. , Richter, E. A. , James, D. E. , Wojtaszewski, J. F. P. , & Febbraio, M. A. (2018). Extracellular vesicles provide a means for tissue crosstalk during exercise. Cell Metabolism, 27(1), 237–251.e4. 10.1016/j.cmet.2017.12.001 29320704

[eph13161-bib-0044] Wu, H. , & Ballantyne, C. M. (2017). Skeletal muscle inflammation and insulin resistance in obesity. Journal of Clinical Investigation, 127(1), 43–54. 10.1172/JCI88880 28045398 PMC5199705

[eph13161-bib-0045] You, T. , Arsenis, N. C. , Disanzo, B. L. , & Lamonte, M. J. (2013). Effects of exercise training on chronic inflammation in obesity: Current evidence and potential mechanisms. Sports Medicine (Auckland, N.Z.), 43(4), 243–256. 10.1007/s40279-013-0023-3 23494259

[eph13161-bib-0046] Zhou, D. , Strakovsky, R. S. , Zhang, X. , & Pan, Y.‐X. (2012). The skeletal muscle Wnt pathway may modulate insulin resistance and muscle development in a diet‐induced obese rat model. Obesity (Silver Spring), 20(8), 1577–1584. 10.1038/oby.2012.42 22349736

