# Supplementary material for: Obesity and exercise training alter inflammatory pathway skeletal muscle small extracellular vesicle microRNAs
Source: Exp Physiol. 2022 Apr 3;107(5):462–75. doi: 10.1113/EP090062 (PMC9323446; doi:10.1113/EP090062)
Supplement: Supplementary file 1 — Statistical Summary Document [file EPH-107-462-s005.docx]

**Manuscript Title:** Obesity and Exercise Training Alter Inflammatory Pathway Skeletal Muscle Small Extracellular Vesicle miRNAs

**Authors:** Brian P. Sullivan, Yaohui Nie, Sheelagh Evans, Chris K. Kargl, Zach R. Hettinger, Ron T. Garner, Monica J. Hubal, Shihuan Kuang, Julianne Stout, Timothy P. Gavin

**Animal model used, if applicable: N/A**

**Underlying hypothesis:** This study investigates the hypothesis that obesity negatively and exercise training beneficially alters skeletal muscle EV miRNA content and inflammatory signalling.

**Definitions of ‘n’:**

n= number of subjects per group.

**Statistical summary table:**

| **Experimental question number** | **Finding/ conclusion** | **Experimental location / variable** | **Mean value** | **Standard Deviation** | **n** | **Exact P value** | **Figure/table in which data are presented** | **Units** | **Data comparisons** | **Statistical test** | **Any other experimental factors** | **Comments** |
| --- | --- | --- | --- | --- | --- | --- | --- | --- | --- | --- | --- | --- |
| 1. Is one week of exercise training sufficient to improve systemic inflammation? | Serum TNF-α is elevated in obese but is not altered with exercise training | Lean Pre | 0.7 | 0.3 | 8 | Interaction (p=0.117) \| Exercise Training (p=0.669) \|  BMI (p=**0.0372**) | Table 1 |  | Lean v Obese \| Pre v Post Exercise Training | 2-way RM ANOVA |  |  |
|  |  | Lean Post | 0.6 | 5 | 8 |  |  |  |  |  |  |  |
|  |  | Obese Pre | 2.6 | 0.2 | 8 |  |  |  |  |  |  |  |
|  |  | Obese Post | 0.9 | 0.2 | 8 |  |  |  |  |  |  |  |
|  | Serum CRP is elevated in obese but is not altered with exercise training | Lean Pre | 1175 | 1135 | 8 | Interaction (p=0.613) \| Exercise Training (p=0.723) \| BMI (p=**0.0001**) |  |  | Lean v Obese \| Pre v Post Exercise Training | 2-way RM ANOVA |  |  |
|  |  | Lean Post | 906 | 371 | 8 |  |  |  |  |  |  |  |
|  |  | Obese Pre | 5284 | 2478 | 8 |  |  |  |  |  |  |  |
|  |  | Obese Post | 5331 | 2039 | 8 |  |  |  |  |  |  |  |
|  | Serum IL-6 is not altered with obesity or exercise training | Lean Pre | 1.4 | 0.7 | 8 | Interaction (p=0.271) \| Exercise Training (p=0.509) \|  BMI (p=0.171) |  |  | Lean v Obese \| Pre v Post Exercise Training | 2-way RM ANOVA |  |  |
|  |  | Lean Post | 1.8 | 2.1 | 8 |  |  |  |  |  |  |  |
|  |  | Obese Pre | 3.7 | 4.6 | 8 |  |  |  |  |  |  |  |
|  |  | Obese Post | 2 | 1.1 | 8 |  |  |  |  |  |  |  |
| 2. RT-PCR on whole muscle homogenates from Lean and obese before and after concurrent exercise training. | Individuals with obesity have reduced expression of IGF-1 mRNA following exercise training | Lean Pre | 1 | 0.52 | 8 | Interaction (p=**0.0061**) | Figure 2 | au | Lean v Obese \| Pre v Post Exercise Training | 2-way RM ANOVA |  |  |
|  |  | Lean Post | 2.24 | 0.76 | 8 |  |  |  |  |  |  |  |
|  |  | Obese Pre | 0.71 | 0.44 | 8 |  |  |  |  |  |  |  |
|  |  | Obese Post | 1.05 | 0.38 | 8 |  |  |  |  |  |  |  |
|  | Individuals with obesity have reduced expression of Wnt 3a mRNA | Lean Pre | 1 | 0.85 | 8 | Interaction (p=0.5887) \| Exercise Training (p=0.2676) \|  BMI (p=**0.0103**) |  | au | Lean v Obese \| Pre v Post Exercise Training | 2-way RM ANOVA |  |  |
|  |  | Lean Post | 1.4 | 0.66 | 8 |  |  |  |  |  |  |  |
|  |  | Obese Pre | 0.59 | 0.22 | 8 |  |  |  |  |  |  |  |
|  |  | Obese Post | 0.79 | 0.49 | 8 |  |  |  |  |  |  |  |
|  | Individuals with obesity have reduced expression of Wnt 5a mRNA | Lean Pre | 1 | 0.41 | 8 | Interaction (p=0.3369) \| Exercise Training (p=0.0651) \|  BMI (p=**0.0109**) |  | au | Lean v Obese \| Pre v Post Exercise Training | 2-way RM ANOVA |  |  |
|  |  | Lean Post | 1.45 | 0.33 | 8 |  |  |  |  |  |  |  |
|  |  | Obese Pre | 0.87 | 0.39 | 8 |  |  |  |  |  |  |  |
|  |  | Obese Post | 1.08 | 0.26 | 8 |  |  |  |  |  |  |  |
|  | Individuals with obesity have reduced expression of Wnt 7a mRNA | Lean Pre | 1 | 0.76 | 8 | Interaction (p=0.8279) \| Exercise Training (p=0.3122) \|  BMI (p=**0.0291**) |  | au | Lean v Obese \| Pre v Post Exercise Training | 2-way RM ANOVA |  |  |
|  |  | Lean Post | 1.27 | 0.54 | 8 |  |  |  |  |  |  |  |
|  |  | Obese Pre | 0.63 | 0.29 | 8 |  |  |  |  |  |  |  |
|  |  | Obese Post | 0.88 | 0.55 | 8 |  |  |  |  |  |  |  |
|  | Exercise Training reduces skeletal muscle IL-8 mRNA | Lean Pre | 1 | 0.74 | 8 | Interaction (p=0.7776) \| Exercise Training (p=**0.0115**) \|  BMI (p=0.3074) | Figure 3 | au | Lean v Obese \| Pre v Post Exercise Training | 2-way RM ANOVA |  |  |
|  |  | Lean Post | 0.57 | 0.2 | 8 |  |  |  |  |  |  |  |
|  |  | Obese Pre | 0.89 | 0.42 | 8 |  |  |  |  |  |  |  |
|  |  | Obese Post | 0.37 | 0.1 | 8 |  |  |  |  |  |  |  |
|  | Exercise Training reduces skeletal muscle IL-10 mRNA | Lean Pre | 1 | 1.01 | 8 | Interaction (p=0.708) \| Exercise Training (p=0.0990) \|  BMI (p=0.2505) |  | au | Lean v Obese \| Pre v Post Exercise Training | 2-way RM ANOVA |  |  |
|  |  | Lean Post | 0.55 | 0.45 | 8 |  |  |  |  |  |  |  |
|  |  | Obese Pre | 0.67 | 0.35 | 8 |  |  |  |  |  |  |  |
|  |  | Obese Post | 0.38 | 0.27 | 8 |  |  |  |  |  |  |  |
|  | Exercise Training reduces skeletal muscle Jun mRNA | Lean Pre | 1 | 0.34 | 8 | Interaction (p=0.8793) \| Exercise Training (p=**0.0062**) \|  BMI (p=0.9691) |  | au | Lean v Obese \| Pre v Post Exercise Training | 2-way RM ANOVA |  |  |
|  |  | Lean Post | 0.75 | 0.18 | 8 |  |  |  |  |  |  |  |
|  |  | Obese Pre | 0.98 | 0.19 | 8 |  |  |  |  |  |  |  |
|  |  | Obese Post | 0.76 | 0.22 | 8 |  |  |  |  |  |  |  |
|  | Exercise Training reduces skeletal muscle Fos mRNA | Lean Pre | 1 | 1.1 | 8 | Interaction (p=0.543) \| Exercise Training (p=**0.0153**) \|  BMI (p=0.5142) |  | au | Lean v Obese \| Pre v Post Exercise Training | 2-way RM ANOVA |  |  |
|  |  | Lean Post | 0.37 | 0.3 | 8 |  |  |  |  |  |  |  |
|  |  | Obese Pre | 1.44 | 1.53 | 8 |  |  |  |  |  |  |  |
|  |  | Obese Post | 0.44 | 0.37 | 8 |  |  |  |  |  |  |  |
