# Supplementary material for: Obesity and exercise training alter inflammatory pathway skeletal muscle small extracellular vesicle microRNAs
Source: Exp Physiol. 2022 Apr 3;107(5):462–75. doi: 10.1113/EP090062 (PMC9323446; doi:10.1113/EP090062)
Supplement: Supplementary file 2 — Table S1. Primer sequences for qRT‐PCR. Table S2. Common differentially expressed skeletal muscle extracellular vesicle miRNAs between individuals with obesity and lean controls following one week of concurrent exercise training. Fold change < 0.8 = down. Fold change > 1.2 = up. ‐ = no change. Table S3. Uncommon differentially expressed skeletal muscle extracellular vesicle miRNAs between individuals with obesity and lean controls following one week of concurrent exercise training. Fold change < 0.8 = down. Fold change > 1.2 = up. ‐ = no change. Table S4. Subject characteristics for lean (LN) vs obese (OB) miRNA SEQ samples. V̇O2MAX ‐ maximal oxygen consumption; 1 RM‐ one repetition maximum; BMI ‐ body mass index; HOMA‐IR ‐ homeostasis model assessment – insulin resistance; HOMA‐β ‐ homeostasis model assessment – β‐cell function; TC – Total Cholesterol; HDL ‐ high density lipoprotein; LDL ‐ low density lipoprotein; TG – Triglycerides; TNF‐α ‐ Tumor Necrosis Factor α; CRP ‐ C‐reactive Protein; IL‐6 ‐ Interleukin‐6. Mean ± SE. Table S5. Subject characteristics for exercise training miRNA SEQ samples. V̇O2MAX ‐ maximal oxygen consumption; 1 RM‐ one repetition maximum; BMI ‐ body mass index; HOMA‐IR ‐ homeostasis model assessment – insulin resistance; HOMA‐β ‐ homeostasis model assessment – β‐cell function; TC – Total Cholesterol; HDL ‐ high density lipoprotein; LDL ‐ low density lipoprotein; TG – Triglycerides; TNF‐α ‐ Tumor Necrosis Factor α; CRP ‐ C‐reactive Protein; IL‐6 ‐ Interleukin‐6. Mean ± SE. [file EPH-107-462-s006.docx]

**Title:** Obesity and Exercise Training Alter Inflammatory Pathway Skeletal Muscle Small Extracellular Vesicle miRNAs

**Authors:** Brian P. Sullivan^1^, Yaohui Nie^1^, Sheelagh Evans^1^, Chris K. Kargl^1^, Zach R. Hettinger^1^, Ron T. Garner^2^, Monica J. Hubal^3^, Shihuan Kuang^1^, Julianne Stout^4^, Timothy P. Gavin^1^ FACSM

**Corresponding Author:** Timothy P. Gavin PhD, Department of Health and Kinesiology, Purdue University, 800 W. Stadium Ave., West Lafayette, IN 47907. Phone: (765) 494-3179, Fax: (765) 496-1239, Email: [gavin1@purdue.edu](mailto:gavin1@purdue.edu)

| **Gene Name** | **Gene ID** | **Forward (5'-3')** | **Reverse (5'-3')** |
| --- | --- | --- | --- |
| 18s | 106632259 | GGCCCTGTAATTGGAATGAGTC | CCAAGATCCAACTACGAGCTT |
| IGF-1 | 3479 | GCTCTTCAGTTCGTGTGTGGA | GCCTCCTTAGATCACAGCTCC |
| β−Catenin | 1499 | AGCTTCCAGACACGCTATCAT | CGGTACAACGAGCTGTTTCTAC |
| Wnt3a | 89780 | AGGAAATTCAGCCCACCAGC | AGGAAATTCAGCCCACCAGC |
| Wnt5a | 7474 | TCGACTATGGCTACCGCTTTG | CACTCTCGTAGGAGCCCTTG |
| Wnt7a | 7576 | CAAGGAGCCGTGTAGAAGTGT | GCAAGGTGGGTACCTGCAA |
| IL-8 | 3576 | TTTTGCCAAGGAGTGCTAAAGA | AACCCTCTGCACCCAGTTTTC |
| IL-10 | 3586 | GACTTTAAGGGTTACCTGGGTTG | TCACATGCGCCTTGATGTCTG |
| Jun | 3725 | TCCAAGTGCCGAAAAAGGAAG | CGAGTTCTGAGCTTTCAAGGT |
| FOS | 2353 | GGGGCAAGGTGGAACAGTTAT | CCGCTTGGAGTGTATCAGTCA |

Supplemental Table 1. Primer sequences for qRT-PCR.

| **Mature MicroRNA** | **Lean Fold Change** | **Lean Change** | **Obese Fold Change** | **Obese Change** |
| --- | --- | --- | --- | --- |
| hsa-let-7f-2-3p | 0.52 | down | 0.71 | down |
| hsa-let-7i-3p | 0.37 | down | 0.71 | down |
| hsa-miR-101-5p | 0.43 | down | 0.56 | down |
| hsa-miR-1301-3p | 2.28 | up | 1.57 | up |
| hsa-miR-1307-3p | 1.83 | up | 1.64 | up |
| hsa-miR-146b-5p | 2.43 | up | 1.51 | up |
| hsa-miR-185-5p | 1.22 | up | 1.66 | up |
| hsa-miR-199a-5p | 0.69 | down | 0.62 | down |
| hsa-miR-199b-5p | 0.76 | down | 0.56 | down |
| hsa-miR-208b-3p | 0.39 | down | 0.71 | down |
| hsa-miR-23a-5p | 3.41 | up | 1.46 | up |
| hsa-miR-296-3p | 2.48 | up | 2.24 | up |
| hsa-miR-3178 | 41.05 | up | 1.31 | up |
| hsa-miR-3609 | 0.38 | down | 0.65 | down |
| hsa-miR-3615 | 1.45 | up | 2.45 | up |
| hsa-miR-370-3p | 2.74 | up | 1.56 | up |
| hsa-miR-3960 | 2.73 | up | 2.00 | up |
| hsa-miR-409-3p | 1.96 | up | 1.76 | up |
| hsa-miR-431-5p | 5.59 | up | 1.43 | up |
| hsa-miR-4485-3p | 28.56 | up | 1.69 | up |
| hsa-miR-4485-5p | 12.98 | up | 1.87 | up |
| hsa-miR-4488 | 11.88 | up | 3.71 | up |
| hsa-miR-4497 | 4.06 | up | 1.80 | up |
| hsa-miR-483-3p | 1.36 | up | 1.77 | up |
| hsa-miR-483-5p | 1.76 | up | 1.82 | up |
| hsa-miR-485-5p | 2.13 | up | 1.34 | up |
| hsa-miR-486-5p | 1.36 | up | 1.33 | up |
| hsa-miR-548h-3p | 0.17 | down | 0.70 | down |
| hsa-miR-548z | 0.17 | down | 0.70 | down |
| hsa-miR-6126 | 4.95 | up | 1.72 | up |
| hsa-miR-629-5p | 2.02 | up | 1.31 | up |
| hsa-miR-675-5p | 4.93 | up | 1.35 | up |
| hsa-miR-7-5p | 1.91 | up | 1.52 | up |

Supplemental Table 2. Common differentially expressed skeletal muscle extracellular vesicle miRNAs between individuals with obesity and lean controls following one week of concurrent exercise training. Fold change <0.8= down. Fold change >1.2= up. - = no change.

| **Mature MicroRNA** | **Lean Fold Change** | **Lean Change** | **Obese Fold Change** | **Obese Change** |
| --- | --- | --- | --- | --- |
| hsa-miR-1-5p | 1.15 | - | 0.36 | down |
| hsa-miR-1291 | 2.44 | up | 1.03 | - |
| hsa-miR-1306-5p | 0.31 | down | 0.92 | - |
| hsa-miR-142-5p | 0.48 | down | 1.79 | up |
| hsa-miR-143-5p | 2.00 | up | 0.49 | down |
| hsa-miR-144-3p | 0.31 | down | 2.77 | up |
| hsa-miR-144-5p | 0.65 | down | 1.86 | up |
| hsa-miR-1469 | 8.82 | up | 0.22 | down |
| hsa-miR-155-5p | 2.90 | up | 1.02 | - |
| hsa-miR-182-5p | 1.09 | - | 2.13 | up |
| hsa-miR-183-5p | 0.87 | - | 2.06 | up |
| hsa-miR-18a-3p | 1.00 | - | 3.92 | up |
| hsa-miR-190a-5p | 0.26 | down | 0.94 | - |
| hsa-miR-30b-5p | 0.90 | - | 0.61 | down |
| hsa-miR-374a-3p | 0.42 | down | 0.91 | - |
| hsa-miR-374b-5p | 0.96 | - | 0.68 | down |
| hsa-miR-4284 | 35.10 | up | 1.12 | - |
| hsa-miR-432-5p | 2.78 | up | 0.96 | - |
| hsa-miR-4492 | 6.04 | up | 1.07 | - |
| hsa-miR-4792 | 4.34 | up | 0.35 | down |
| hsa-miR-6087 | 9.00 | up | 0.87 | - |
| hsa-miR-6724-5p | 116.62 | up | 0.81 | - |
| hsa-miR-7641 | 16.23 | up | 0.48 | down |
| hsa-miR-7704 | 21.43 | up | 1.04 | - |
| hsa-miR-885-5p | 0.44 | down | 1.25 | up |

Supplemental Table 3. Uncommon differentially expressed skeletal muscle extracellular vesicle miRNAs between individuals with obesity and lean controls following one week of concurrent exercise training. Fold change <0.8= down. Fold change >1.2= up. - = no change.

|  | **LN (n=3)** | **OB (n=4)** | **p-value** |
| --- | --- | --- | --- |
| **Age** | 28.7 ± 2.4 | 26.8 ± 2.3 | 0.59 |
| **Height (m)** | 1.74 ± 0.07 | 1.72 ± 0.06 | 0.80 |
| **Weight (kg)** | 68.3 ± 5.3 | 95.7 ± 8.7 | 0.06 |
| **BMI (kg/m^2^)** | 22.4 ± 0.5 | 32.2 ± 1.0 | <0.01 |
| **VO_2_max (L/min)** | 2.21 ± 0.49 | 2.34 ± 0.34 | 0.82 |
| **VO_2_max (ml/kg/min)** | 31.6 ± 4.4 | 23.8 ± 3.4 | 0.21 |
| **1 RM (kg)** | 113.6 ± 22.7 | 164.4 ± 42.5 | 0.39 |
| **1 RM (kg/kg)** | 1.63 ± 0.20 | 1.66 ± 0.34 | 0.96 |
| **Glucose (mg/dl)** | 82.7 ± 5.0 | 84.0 ± 6.9 | 0.89 |
| **Insulin (uIU/ml)** | 10.0 ± 1.0 | 26.0 ± 9.3 | 0.21 |
| **HOMA-IR** | 2.02 ± 0.1 | 5.37 ± 1.9 | 0.20 |
| **HOMA-β** | 218.4 ± 74.6 | 770.0 ± 330.0 | 0.22 |
| **TC (mg/dL)** | 204.3 ± 13.9 | 201.8 ± 24.6 | 0.94 |
| **HDL (mg/dL)** | 55.0 ± 2.1 | 42.0 ± 4.7 | 0.08 |
| **LDL (mg/dL)** | 125.0 ± 14.0 | 129.8 ± 23.8 | 0.88 |
| **TG (mg/dL)** | 121.3 ± 16.0 | 149.5 ± 21.6 | 0.37 |
| **TNF-⍺ (pg/µL)** | 0.73 ± 0.17 | 0.87 ± 0.05 | 0.38 |
| **CRP (pg/µL)** | 1440.4 ± 1075.7 | 3604.8 ± 932.7 | 0.19 |
| **Il-6 (pg/µL)** | 1.85 ± 0.54 | 2.94 ± 0.48 | 0.19 |

Supplemental Table 4. Subject characteristics for lean (LN) vs obese (OB) miRNA SEQ samples. V̇O_2MAX_ - maximal oxygen consumption; 1 RM- one repetition maximum; BMI - body mass index; HOMA-IR - homeostasis model assessment – insulin resistance; HOMA-β - homeostasis model assessment – β-cell function; TC – Total Cholesterol; HDL - high density lipoprotein; LDL - low density lipoprotein; TG – Triglycerides; TNF-⍺ - Tumor Necrosis Factor ⍺; CRP - C-reactive Protein; IL-6 - Interleukin-6. Mean ± SE.

|  | **Pre (n=5)** | **Post (n=5)** | **p-value** |
| --- | --- | --- | --- |
| **Age** | 28.4 ± 1.6 | - | - |
| **Height (m)** | 1.73 ± 0.06 | - | - |
| **Weight (kg)** | 86.1 ± 9.1 | 85.2 ± 8.4 | 0.27 |
| **BMI (kg/m^2^)** | 28.7 ± 2.5 | 28.4 ± 2.4 | 0.25 |
| **VO_2_max (L/min)** | 2.21 ± 0.27 | - | - |
| **VO_2_max (ml/kg/min)** | 25.8 ± 3.9 | - | - |
| **1 RM (kg)** | 134.8 ± 29.9 | - | - |
| **1 RM (kg/kg)** | 1.53 ± 0.21 | - | - |
| **Glucose (mg/dl)** | 78.6 ± 3.85 | 86.6 ± 1.5 | 0.10 |
| **Insulin (uIU/ml)** | 22.6 ± 8.0 | 22.6 ± 9.6 | 1.00 |
| **HOMA-IR** | 4.5 ± 1.7 | 4.8 ± 2.0 | 0.92 |
| **HOMA-β** | 700.6 ± 262.1 | 349.1 ± 151.3 | 0.11 |
| **TC (mg/dL)** | 215.6 ± 15.8 | 208.0 ± 21.1 | 0.52 |
| **HDL (mg/dL)** | 48.6 ± 4.8 | 50.4 ± 6.7 | 0.66 |
| **LDL (mg/dL)** | 137.2 ± 17.4 | 137.0 ± 20.7 | 0.98 |
| **TG (mg/dL)** | 148.8 ± 16.6 | 104.0 ± 13.9 | 0.07 |
| **TNF-⍺ (pg/µL)** | 0.78 ± 0.1 | 0.93 ± 0.1 | 0.05 |
| **CRP (pg/µL)** | 3480.3 ± 825.7 | 3108.1 ± 806.0 | 0.56 |
| **Il-6 (pg/µL)** | 2.9 ± 0.4 | 1.91 ± 0.5 | 0.04 |

Supplemental Table 5. Subject characteristics for exercise training miRNA SEQ samples. V̇O_2MAX_ - maximal oxygen consumption; 1 RM- one repetition maximum; BMI - body mass index; HOMA-IR - homeostasis model assessment – insulin resistance; HOMA-β - homeostasis model assessment – β-cell function; TC – Total Cholesterol; HDL - high density lipoprotein; LDL - low density lipoprotein; TG – Triglycerides; TNF-⍺ - Tumor Necrosis Factor ⍺; CRP - C-reactive Protein; IL-6 - Interleukin-6. Mean ± SE.
